# Novel Salinity Tolerance Loci in Chickpea Identified in Glasshouse and Field Environments

**DOI:** 10.3389/fpls.2021.667910

**Published:** 2021-04-28

**Authors:** Judith Atieno, Timothy D. Colmer, Julian Taylor, Yongle Li, John Quealy, Lukasz Kotula, Dion Nicol, Duong T. Nguyen, Chris Brien, Peter Langridge, Janine Croser, Julie E. Hayes, Tim Sutton

**Affiliations:** ^1^South Australian Research and Development Institute, Adelaide, SA, Australia; ^2^School of Agriculture, Food and Wine, University of Adelaide, Adelaide, SA, Australia; ^3^School of Agriculture and Environment, The University of Western Australia, Perth, WA, Australia; ^4^Department of Primary Industries and Regional Development, Dryland Research Institute, South Perth, WA, Australia; ^5^The Plant Accelerator, Australian Plant Phenomics Facility, University of Adelaide, Adelaide, SA, Australia

**Keywords:** chickpea, salt stress, tissue Na^+^, multiple environment phenotyping, linkage mapping, QTL, salt tolerance, accelerated-Single Seed Descent

## Abstract

A better understanding of the genetics of salinity tolerance in chickpea would enable breeding of salt tolerant varieties, offering potential to expand chickpea production to marginal, salinity-affected areas. A Recombinant Inbred Line population was developed using accelerated-Single Seed Descent of progeny from a cross between two chickpea varieties, Rupali (salt-sensitive) and Genesis836 (salt-tolerant). The population was screened for salinity tolerance using high-throughput image-based phenotyping in the glasshouse, in hydroponics, and across 2 years of field trials at Merredin, Western Australia. A genetic map was constructed from 628 unique *in-silico* DArT and SNP markers, spanning 963.5 cM. Markers linked to two flowering loci identified on linkage groups CaLG03 and CaLG05 were used as cofactors during genetic analysis to remove the confounding effects of flowering on salinity response. Forty-two QTL were linked to growth rate, yield, and yield component traits under both control and saline conditions, and leaf tissue ion accumulation under salt stress. Residuals from regressions fitting best linear unbiased predictions from saline conditions onto best linear unbiased predictions from control conditions provided a measure of salinity tolerance *per se*, independent of yield potential. Six QTL on CaLG04, CaLG05, and CaLG06 were associated with tolerance *per se*. In total, 21 QTL mapped to two distinct regions on CaLG04. The first distinct region controlled the number of filled pods, leaf necrosis, seed number, and seed yield specifically under salinity, and co-located with four QTL linked to salt tolerance *per se*. The second distinct region controlled 100-seed weight and growth-related traits, independent of salinity treatment. Positional cloning of the salinity tolerance-specific loci on CaLG04, CaLG05, and CaLG06 will improve our understanding of the key determinants of salinity tolerance in chickpea.

## Introduction

Salinity is an abiotic stress which has a negative impact on crop productivity (Rengasamy, 2006; Nawaz et al., 2010). Grain legumes are generally sensitive to salinity, with faba bean, field pea, and chickpea being the most sensitive (Maas and Hoffman, 1977). Chickpea is the third most cultivated legume globally (FAO, 2019) and sensitive genotypes are impacted negatively in as little as 25 mM NaCl in hydroponics experiments (Flowers et al., 2010). Chickpea is most sensitive at reproductive stage (Vadez et al., 2007, 2012b; Krishnamurthy et al., 2011; Samineni et al., 2011).

Phenotyping for salinity tolerance is difficult as it is an environmentally and developmentally regulated trait. Several studies have aimed to understand further the complexity of salinity tolerance in chickpea from both physiological and genetics perspectives (Vadez et al., 2007, 2012a,b; Turner et al., 2013; Khan et al., 2015, 2016; Pushpavalli et al., 2015a,b; Atieno et al., 2017; Kotula et al., 2019). Tolerance in controlled conditions may not translate to meaningful tolerance in the field (Tavakkoli et al., 2012) and similarly, tolerance at vegetative stage of development may not be expressed during reproductive stage (Vadez et al., 2007). Therefore, it is imperative to phenotype plants at different developmental stages and under different environments to make accurate inferences regarding the tolerance status of different genotypes.

Because of the inherent complexity of salinity tolerance in plants, the application of reliable and relevant phenotyping methodologies is critical. There are a variety of phenotyping platforms each with different merits which need to be considered before setting up an experiment. Image-based phenotyping under controlled conditions can detect subtle differences between plants without bias and eliminate confounding variability that is typical in the field. Field phenotyping while challenging due to spatial and temporal variability of salinity and other soil parameters is required when conducting research of agronomic and breeding relevance. To counteract this variability, studies such as Saade et al. (2016) and Saade et al. (2020) irrigated sandy experimental field sites with saline water to achieve uniform field saline levels. Other studies in different crops have combined high throughput image-based phenotyping with field phenotyping to dissect the genetic components of different traits; as examples, growth and transpiration under water-deficit in wheat (Parent et al., 2015) and genetic components of salinity tolerance in barley (Saade et al., 2020). To the best of our knowledge, this is the first study to combine high throughtput image-based phenotyping and field phenotyping to investigate the genetics of salinity tolerance in chickpea.

Linkage mapping has been utilized to identify genomic regions underlying salinity tolerance related traits in chickpea (Samineni, 2010; Vadez et al., 2012a; Pushpavalli et al., 2015a; Soren et al., 2020). Using linkage maps with low marker density, Vadez et al. (2012a) and Pushpavalli et al. (2015a) identified QTLs within large intervals associated with seed yield and yield related traits under saline conditions. Most recently, utilizing a higher density genetic map, Soren et al. (2020) identified QTL for yield and yield related traits for chickpea under salinity. However, whilst relevant, these studies focussed on Indian adapted varieties and it has not been established if tolerance mechanisms expressed in these genotypes in India are relevant in Australian conditions. Chickpea breeding in Australia has historically utilized a source of salinity tolerance derived from the desi variety Genesis836, adapted to Australian conditions. However, the difficulties associated with phenotyping for salinity tolerance-related traits and the lack of molecular markers to select for salinity tolerance has limited progress in improving tolerance in the breeding program (Kristy Hobson, chickpea breeder, Pers. Com.).

The influence of maturity and flowering on the expression of salinity tolerance in plants needs to be allowed for during analysis. Salinity has been reported to delay the time to flower and maturity in chickpea (Krishnamurthy et al., 2011; Vadez et al., 2012b; Pushpavalli et al., 2015b). Vadez et al. (2007) described a relationship between days to flower and seed yield with very early and late maturing genotypes having increased sensitivity to salt. Although this phenomenon was not observed in all studies (e.g., Krishnamurthy et al., 2011; Turner et al., 2013; Atieno et al., 2017), flowering time requires consideration during genetic analyses to remove its confounding effect and allow for accurate detection of QTLs associated with salinity tolerance. Vadez et al. (2012a) conducted separate analyses within early and late flowering groups in a population developed between JG 62 (tolerant) and ICCV 2 (sensitive) chickpea genotypes segregating for flowering time to reduce the confounding effect of flowering. This approach reduces sample size and thus lowers the power and reliability of detecting QTLs. This study has utilized a better strategy of controlling flowering time by incorporating flowering loci as cofactors during genetic analysis in chickpea.

Conventionally, salinity tolerance is defined as the ratio of measurements obtained from genotypes under salt-stressed conditions against measurements of the same genotypes grown in control conditions. Ratio data, however, may depart significantly from normality and typically requires transformation before downstream analysis (Curran-Everett, 2013). To circumvent these challenges, this study has used residuals from a regression line fitting best linear unbiased predictors (BLUPs) from saline conditions on to BLUPs from control conditions to provide a measure of salinity tolerance *per se*, that is independent of yield potential. This method has also been used by Vadez et al. (2007), and most recently by Temme et al. (2020) to define salinity tolerance.

By utilizing different phenotyping platforms, accounting for flowering and adopting an alternative approach to quantify salinity tolerance, the present study used a Rupali/Genesis836 recombinant inbred line (RIL) population, developed using accelerated-Single Seed Descent methodology, to understand the physiological and genetic mechanisms of salinity tolerance in Australian germplasm. The ultimate aim is to improve the accuracy and rate of genetic gain in developing saline-tolerant chickpea varieties.

## Materials and Methods

### Plant Material

#### Recombinant Inbred Line (RIL) Population

A RIL population consisting of 200 lines was developed from a cross between two desi Australian adapted chickpea (*Cicer arietinum*) varieties, Rupali and Genesis836, previously shown to contrast for salinity tolerance (Turner et al., 2013; Khan et al., 2015). Genesis836 is a direct introduction from the International Centre for Research in the Semi-Arid Tropics (ICRISAT, India) and has been utilized as a source of salinity tolerance in the Australian chickpea breeding program. Rupali was bred by the Department of Agriculture and Food Western Australia (DAFWA) and the Centre for Legumes in Mediterranean Agriculture (CLIMA), based at the University of Western Australia (Clarke et al., 2004). Seed of cv. Rupali and cv. Genesis836 were obtained from the Australian Grains Genebank (AGG). One of the F1 plants derived from a Rupali by Genesis836 cross was vegetatively propagated to achieve high F2 seed numbers (as per Danehloueipour et al., 2006). The F_2_ seed was sent to The University of Western Australia (31.9800° S, 115.8190° E) for rapid RIL development. The F_2:5_ were individually tracked and cycled under accelerated-Single Seed Descent (aSSD) conditions modified from Croser et al. (2016) and Ribalta et al. (2017). Two seeds were sown into each 0.4 L pot containing pinebark: peat: sand (2.5:1:1.5) potting mix (Richgro Garden Products) at pH 6.5, grown at 22°C day/18°C night (±1°C), 20 h photoperiod and RH 70 ± 10% and thinned on emergence to one seedling/pot. Plants were hand watered daily and fertilized weekly with N:P:K fertilizer. Light was provided solely by Valoya AP67 Series B light emitting diode based arrays, with red:far-red ratio of 2.89 and intensity of c. 325 μmol m^−2^ s^−1^ at canopy (Valoya Oy, Helsinki, Finland). Light spectra and intensity were measured using a Sekonic C7000 SpectroMaster spectrometer (Sekonic Corp., Tokyo, Japan) and calculation of spectral ratio was as Runkle and Heins (2001). Flowering was recorded across all lines within 23–28 d of sowing and immature seed was removed at physiological maturity (*c*. 18 days after flowering) and resown to the following generation. Taking T_b_ to be 0°C (as per Lake et al., 2016), growing degree days from sowing to harvest = 874–981. The F_4:5_ seed was left to fully mature on the plant and 3–10 seed from each RIL returned to University of Adelaide for phenotypic and genotypic characterization.

### Phenotypic Evaluation in the Glasshouse

Phenotyping of the Rupali/Genesis836 RIL population (*n* = 200) was conducted in The Plant Accelerator located at the Waite Campus of the University of Adelaide (http://www.plantphenomics.org.au/services/accelerator/), as described in Atieno et al. (2017) with minor modifications. The experiment was conducted between June and November 2015. The glasshouse temperature and humidity were controlled and ranged from 24 ± 2°C/40% (day) to 16 ± 2°C/90% (night), respectively. The experiment was set up in two smarthouses (growth rooms) utilizing 20 lanes and 22 positions. Each RIL was replicated twice while the parents were replicated 10 times in a design described in Atieno et al. (2017). Four hours of supplemental lighting was provided in growth rooms to extend daylight to 12 h. Plants were first imaged at 30 days after sowing (DAS) for 3 days prior to salt application to quantify plant growth rate before salt application. At 33 DAS, each pot received either 0 or 70 mM NaCl, equivalent to applying 100 ml of 0 or 250 mM NaCl, respectively. To maintain salt concentration in the pots, all pots were watered and maintained at field capacity (17% (w/w), determined gravimetrically). Plants were imaged for a further 13 days to quantify growth under both control and saline conditions. A total of 14,080 visible light (RGB) images were obtained and processed in LemnaGrid (LemnaTec, Germany) to compute projected shoot area (PSA). Relative growth rates (RGR) were computed from smoothed cubic splines fitted for each cart (pot) to the observed PSA for each day of imaging. The difference in the logarithms of the smoothed projected shoot area for two consecutive days of imaging was divided by the number of days between imaging to constitute RGR. In addition to measurements extracted from high-resolution imaging, other measurements included days to first flower, leaf sodium (Na^+^) and potassium (K^+^) content in the youngest fully expanded leaves, final plant height, yield and yield components including shoot biomass at maturity, seed number, total pod number, empty pod number, filled pod number, and 100-seed weight. To investigate the effect of photoperiod on flowering, Genesis836 and Rupali were grown under 3 different light regimes (8, 12, and 16 h) in a temperature and light controlled chamber. Similarly, selected genotypes replicated four times from the RIL population with relatively early (3 genotypes) and late (2 genotypes) flowering times under 12 h of light were compared under 16 h of light.

### Phenotypic Evaluation in the Field

Field trials were conducted at the Department of Primary Industries and Regional Development (DPIRD) research station in Merredin (31.48° S, 118.27° E), Western Australia for two consecutive years in 2017 (Merredin2017) and 2018 (Merredin2018) on two different sites (each with a Merredin Sandy Loam; Bettenay, 1960). A split-plot experimental design was used in each year, with four non-saline (control) and four artificially salinized (NaCl added; see below) blocks. In 2017, each block consisted of 228 rows (214 genotypes (198 RILs) with two parents, Rupali and Genesis836, replicated 4 times in each block, plus border rows). In 2018, each block consisted of 208 rows (198 genotypes (181 RILs) with two parents, Rupali and Genesis836, replicated 4 times in each block, plus 4 border rows). Rows in each block were arranged in 3 columns (76 rows in each column) 0.5 m apart in Merredin2017 or 2 columns (104 rows in each column) 0.4 m apart in Meredin2018. Within each column, each row was 1.25 m long at 0.25 m spacing so that each block was 20.05 m long and 4.75 m wide in Merredin2017 or 28.45 m long and 2.9 m wide in Merredin2018. Genotypes were assigned randomly to each row within each block and 15 seeds were hand planted equally-spaced along the row and at a depth of 30 mm, on 8/9/10 May in 2017, and 22/23/24 May in 2018. A 2.05 m buffer strip of machine-sown (knife points and press wheels, 2.5 cm sowing depth) chickpea (PBA Striker) surrounded each block.

Seeds were treated with P-Pickel T fungicide prior to sowing. Knife points with press wheels were used to create rows at 25.4 cm spacings and with 100 kg ha^−1^ of a granular compound fertilizer (10.2% N, 13.1% P, 10.3% K, 6.7% S, 0.11% Cu, 0.23% Zn, 0.01% Mn; Gusto Gold, Summit Fertilizers) and 10 kg ha^−1^ of granules carrying rhizobia inoculant (Group N, ALOSCA). Application of pre-emergent herbicides achieved weed control and the few weeds present were removed by hand. Prophylactic fungicide sprays to foliage prevented the possibility of any ascochyta blight damage. A precautionary application of insecticide during podding prevented the possibility of pod-borer.

The 2017 season had lower than usual rainfall (Supplementary Figure 1A) and so supplementary irrigation was applied via Trickle-Tape positioned along each row of Merredin2017 with the following amounts and dates: 19 mm, 5 June; 19 mm, 20 June; 10 mm, 15 July; 10 mm, 1 August; 15 mm, 17 September. Merredin2018 was rain fed only (Supplementary Figure 1B).

To impose the salt treatment in 2017, 4 M NaCl solution was applied to all inter-row spaces in each artificially salinized block by using a hooded spray wand connected to a pressurized backpack with a calibrated flow and the operator walking at a known speed. The NaCl applied in 2017 was 28–32 g m^−2^ (total plot area) at 35 DAS and again at 36 DAS, 25–28 g m^−2^ at 64 DAS and again at 65 and 85 DAS, 28–31 g m^−2^ at 86 DAS, 24–25 g m^−2^ at 95 DAS, and 21–24 g m^−2^ at 96 DAS. In 2018, NaCl was applied at 75 g m^−2^ prior to sowing (salt broadcast by hand) followed by five additional applications (using the backpack sprayer as described for 2017) of 52–69 g m^−2^ each time at 43/44, 70, 76, 97/98, and 100/101 DAS. Soil salinity was measured at several times across the growing season using an EM-38 meter (Geomatrix Ltd., Leighton Buzzard, UK) and by taking soil cores at locations selected based on EM-38 readings: from the lowest to the highest values with the constraint of 5 locations per block. The soil core samples were taken with a soil auger at depths of 0–10, 10–20, and 20–30 cm, oven dried, and electrical conductivity (EC) and Na^+^, K^+^, and Cl^−^ were measured in 1:5 soil:water extracts. Plant measurements taken were: emergence (2017), days to flowering and podding (2017 and 2018), proportion of leaf necrosis for the whole shoot (1-no symptoms, to 9-all leaves dead) (2017 and 2018), leaf tissue Na^+^, K^+^ and Cl^−^ (2017 and 2018), plant height (2017), above ground biomass (2017 and 2018), number of pods (2017), number of seeds (2017), 100-seed weight (2017), and seed yield (2017 and 2018). The tissue ion analyses were of the youngest fully-expanded leaves taken from two plants in each row at the early podding stage. The leaves were oven-dried, weighed, extracted in 0.5 M HNO_3_, and extracts were analyzed for Na^+^ and K^+^ using a flame photometer and Cl^−^ using a chloridometer (cf. Munns et al., 2010). At maturity, 182 DAS in 2017 and 174 DAS in 2018, plants were counted in each row and the nine inner plants were harvested by cutting the plants at ground level. In 2017, the empty- and filled-pod numbers and seed numbers were recorded after hand threshing. In 2018, plants were mechanically threshed to reduce sample processing time. In both 2017 and 2018, the dry weights of seeds were recorded after drying in a forced-draft oven at 30°C for 72 h, the dry weights of the remaining pod shells, leaves and stems were recorded after oven-drying at 60°C for 48 h.

### Phenotypic Evaluation Under Hydroponics

Supported hydroponics set-up mimicking the screening methodology routinely used in the breeding program was used to assess the salinity tolerance of the Rupali/Genesis836 population. The experimental setup included 181 RILs in addition to Rupali and Genesis836 randomized within three flood-and-drain trays, each measuring 1,040 mm by 2,040 mm. Genotypes were replicated twice in a randomized complete block design. Pots (800 ml) were filled with diatomaceous rocks (5–15 mm diam) and placed in the trays, that were supplied with nutrient solution (pH 6.5) previously used for chickpea (Khan et al., 2016). Chickpea seeds were imbibed in water for ~2 h in the fridge (4°C) prior to sowing. Each pot had four seeds, and after 2.5 weeks, was thinned to one per pot. At 3 weeks, NaCl was added to the nutrient solution to 25 mM, with a second addition made 12 h later to 50 mM, and a third addition 6 h after the second to bring the final NaCl concentration to 80 mM NaCl. The nutrient solution was replaced on a weekly basis and the NaCl concentration maintained during the experimental period. Leaf damage was assessed visually using a necrosis score scale (1–9) at 2 and 3 weeks after NaCl application. Plants were harvested four weeks after NaCl application, dried in a 65°C oven for 48 h and biomass measurements obtained.

### Genotyping

DNA was extracted from 200 RILs and parents (Rupali and Genesis836) using a DNeasy Plant Mini kit (QIAGEN). The quantity and quality of DNA was assessed with a spectrophotometer (ND-100, Biolab). Genotyping was performed using DArTseq (Diversity Array's Technology Pty Ltd, Canberra, Australia) (http://www.diversityarrays.com/dart-application-dartseq) as well as KASP-based genotyping assay (He et al., 2014) using SNPline PCR Genotyping System (LGC, Middlesex, UK). Sequence details of DArT and SNP markers are listed in Supplementary Table 1.

### Linkage Map Construction

The linkage map of the Rupali/Genesis836 RIL population was constructed using a synergistic combination of qtl (Broman and Sen, 2009; Broman and Wu, 2016) and ASMap (Taylor and Butler, 2017) R packages available in the R Statistical Computing Environment (R Core Team, 2020). Preceding linkage map construction, the genetic marker set was diagnostically analyzed. This included a dissimilarity analysis of the progeny to determine their relatedness. Individuals sharing more than 90% of alleles across the marker set were deemed to be genetic clones and used to form consensus genotypes. Marker quality was refined through the removal of markers with <80% observed allelic information. Additionally, markers were removed if they exhibited significant segregation distortion greater than a Bonferroni corrected *p*-value for a familywise alpha level equal to 0.05. The remaining set of markers was clustered into nine linkage groups and the markers were optimally ordered within each linkage group using the MSTMap algorithm (Wu et al., 2008) functionality available in the ASMap package. A graphical diagnosis of recombination and double recombinations of the genotypes for the initial constructed map was performed and genotypes with excessive double recombinations were removed from further linkage map construction. After removal, markers within each linkage group were reordered and a simultaneous graphical examination of the marker and interval profiles was conducted. This identified several markers with excessive double recombinations and these were removed. The markers within linkage groups were optimally ordered a final time and the identification and orientation of the linkage groups occurred through marker sequence comparison with the Kabuli reference assembly v.1 (Varshney et al., 2013) using the BLAST portal https://blast.ncbi.nlm.nih.gov/Blast.cgi. The linkage map was prepared for analysis, by condensing each co-locating set of markers to form a representative consensus marker with a unique map position. Alleles were numerically encoded (AA = 1, BB = −1) and the remaining missing allele calls were numerically imputed using the flanking marker rules of Martinez and Curnow (1992). This complete marker information was then used to calculate pseudo mid-point markers using the formulae derived in Verbyla et al. (2007).

### Phenotypic Linear Mixed Model Analysis

Phenotypic traits of the RIL population from the glasshouse and two field experiments were analyzed using a linear mixed model (LMM) that appropriately partitioned and accounted for genetic and non-genetic sources of variation (Gilmour et al., 1997). Where necessary, traits were transformed to satisfy modeling assumptions. For each of the traits, the LMM consisted of set of fixed effects to capture the estimation of an overall trait mean effect for the RIL lines, and the population parents, independently for the salt and control treatments. Additionally, the fixed effects were also used to model the variation from the physical structures of the design including the different smarthouses in the Plant Accelerator, blocks in the field and hydroponics trays as well as model linear spatial trend across rows within each smarthouse, field block and hydroponics tray. To ensure the underlying genetic variation of the traits were adjusted for flowering time, the fixed component of the LMM contained numerical covariates of two flowering loci modeled independently for each treatment. Additional extraneous non-genetic variation arising from the experiment including variation from zones in the smarthouse and non-linear trends across the ranges within smarthouses and rows in the field was modeled in the LMM using random effects. The model residuals were appropriately partitioned to ensure individual residual variances were estimated for each of the treatments. After initial diagnostic assessment, residual outliers were detected and downweighted with separate random covariates as per Gumedze et al. (2010). To initially test the significance of the genotype by treatment interaction, the LMM was fitted with a fixed component containing a genotype term consisting of a factor with a level for each RIL, a treatment term and genotype by treatment interaction term. The significance of individual terms was then tested using the appropriate Wald statistics and summarized.

To further understand the genetic variation associated with each treatment and to provide a baseline model for the whole genome analysis, an alternative LMM was fitted. The model contained all the terms defined above with the genotype and treatment terms moved from the fixed component of the LMM to a single genotype term appropriately partitioned by treatment to ensure separate genetic variances were estimated for the control and salt treatments. From this fitted LMM, best linear unbiased predictors (BLUPs) of the individual RILs for each treatment trait were extracted and used to perform pairwise correlation analysis. The LMM was then extended to include a parameter to estimate a model based genetic correlation between the salt and control treatments. From this extended fitted LMM, salinity tolerance was obtained from the residuals of a random regression of the best linear unbiased prediction (BLUPs) from saline conditions on to BLUPs from control conditions (McDonald et al., 2015; Mahjourimajd et al., 2016). The regression slope represents the average genetic tolerance of the trait in both saline and control conditions. Regression residuals provide a measure of trait-based salinity tolerance *per se* independent of yield potential where lines with positive residuals represent better than average salinity tolerance and lines with negative residuals poorer than average tolerance to salinity. The Heritability (H^2^) in each of the experimental environments was estimated using Cullis et al. (2006).

All LMM phenotypic analyses of the RIL population were computationally conducted in ASReml-R (Butler et al., 2018) available as a package in the R statistical computing environment R. The package contains a suite of flexible functions for the fitting and diagnosing of complex LMMs and uses the REML algorithm of Patterson and Thompson (1971) to estimate model parameters. The package is available for download from VSN International (https://www.vsni.co.uk). Additional model diagnosis was conducted using the functionality of the ASExtras R package available from http://www.mmade.org.

### Whole Genome QTL Analysis

The whole genome average interval mapping (WGAIM) approach of Verbyla et al. (2007) and Verbyla et al. (2012) was used for detection and summary of QTL for the control, salinity and tolerance traits measured in the RIL population. The WGAIM approach uses an extension of LMMs by incorporating the complete set of linkage map intervals into the random component of the LMM as a single term containing a contiguous block of covariates with a single additive genetic variance parameter. The inclusion of this term is then tested and if found to be significant at an alpha level equal to 0.05, an outlier detection method is used to select a putative interval QTL. The selected QTL is then moved to the fixed component of the LMM and an exclusion window is placed on the left right flanking markers within 20 cM of the QTL. This forward selection process is repeated until the term containing the reduced set of linkage map intervals is non-significant. The selected set of additive QTL intervals are then summarized with their flanking markers, interval distance, size of the putative QTL effect, contribution to genetic variance and LOD score. All QTL analyses and summaries were performed using the wgaim R package (Taylor and Verbyla, 2011) available in the R statistical computing environment.

## Results

### Impact of Salinity on Seed Yield and Yield Components

The salinity levels used in the glasshouse pot assays, hydroponics system and Merredin2017 and Merredin2018 were sufficient to observe an impact on different measurements under salinity relative to control treatments. However, it is important to note the EC values and Na^+^ levels in soil cores were higher in Merredin2018 compared to 2017 (Supplementary Figures 2, 3). Information on the progressions of EC, Na^+^, and Cl^−^ levels in the field are available in Supplementary Figures 2–4.

Means of most traits measured for RILs were within the range of Rupali and Genesis836 means, with the exception of plant height, plant biomass and days to flower in the glasshouse and seed yield in Merredin2018, where the RILs displayed significant transgressive phenotypes compared to the parents (Tables 1–3, Supplementary Table 2). This phenomenon is not surprising, as the RILs are comprised of different genetic composition independently inherited from the parents and thus new genetic recombinations may lead to positive or negative interaction between loci. Significant genotype by treatment interaction (G × T) was observed for most traits measured in the glasshouse (Table 1), with the exception of 100-seed weight, plant height, projected shoot area, relative growth rate, days to flower, K^+^, water use and water use efficiency. In contrast, significant G × T was only observed for necrosis and Cl^−^ content in Merredin2017 (Table 2) and necrosis, Na^+^ content, K^+^:Na^+^ ratio and harvest index in Merredin2018 (Table 3). Significant G × T was observed for all traits measured under hydroponics conditions (Supplementary Table 2). In the instances where G × T was not significant, genotype and/or treatment effects were significant (< 0.001) with the exception of K^+^ content in Merredin2017 (Tables 1–3).

**Table 1:** Summary of measurements taken in the glasshouse under salt and control conditions.

**Traits**	**Treatment**	**Genesis836**	**Rupali**	**Mean**	**Min**	**Max**	**G**	**T**	**G × T**	**Heritability** ** (H^2^)**
Seed yield (g)	Control	6.7	5.1	4.8	1	8				0.47
	Salt	4.4	2.5	4	0.7	7.4	n.a	n.a	<0.001	0.52
Seed number	Control	32	37	27	8	49				0.50
	Salt	26	24	26	4	48	n.a	n.a	<0.001	0.43
100-seed weight (g)	Control	21.3	13.9	19.3	10.6	27.9				0.63
	Salt	17.1	10	14.9	5.1	25.1	<0.001	<0.001	0.67	0.66
Number of total pods	Control	30	32	25	5	42				0.55
	Salt	24	25	24	8	45	n.a	n.a	<0.001	0.41
Number of filled pods	Control	27	28	21	7	41				0.50
	Salt	22	19	19	3	35	n.a	n.a	<0.001	0.41
Number of empty pods	Control	4	4	5	0	15				0.23
	Salt	2	6	3	0	13	n.a	n.a	0.024	0.00
Harvest index	Control	0.44	0.32	0.31	0.06	0.51				0.54
	Salt	0.45	0.3	0.36	0.18	0.5	n.a	n.a	<0.001	0.37
Relative growth rate (pixels pixel^−1^day^−1^)	Control	0.07	0.07	0.08	0.05	0.1				0.00
	Salt	0.06	0.05	0.06	0.04	0.09	<0.001	<0.001	0.29	0.00
Plant height (cm)	Control	53.8	52.8	61.7	35	86.4				0.65
	Salt	41.6	41.2	51.5	28	72.4	<0.001	<0.001	0.645	0.67
Shoot biomass (g)	Control	8.5	10.6	11.3	2.2	21.4				0.51
	Salt	5.2	5.6	7.2	2.2	13.2	n.a	n.a	<0.001	0.45
Projected shoot area (pixels)	Control	3.12×105	3.38 × 105	3.22 × 105	1.29 × 105	5.17 × 105				0.71
	Salt	2.72 × 105	2.90 × 105	2.78 × 105	1.24 × 105	4.87 × 105	<0.001	<0.001	0.159	0.72
Water use (ml)	Control	43.2	42.6	43.9	18.6	65				0.31
	Salt	26.9	29.3	28.5	12.8	46.3	<0.001	<0.001	0.242	0.35
Water use efficiency (pixels ml^−1^)	Control	7.67 × 103	7.20 × 103	7.49 × 103	2.87 × 103	1.13 × 104				0.14
	Salt	1.04 × 104	1.02 × 104	1.05 × 104	5.49 × 103	1.68 × 104	<0.001	<0.001	0.263	0.16
Potassium (μmol g^−1^ DW)	Control	982	969	905.9	519	1,554				0.26
	Salt	1,361	1,374	1329.7	795	2,549	<0.001	<0.001	0.283	0.25
Sodium (μmol g^−1^ DW)	Control	5.2	9.6	8.43	5.61	80.32				0.35
	Salt	49.9	193.4	78.1	2.5	452.3	n.a	n.a	<0.001	0.29
Potassium: Sodium ratio	Control	189.9	101.3	107	1.6	980.9				0.32
	Salt	27.3	7.1	17.2	3.6	148.6	n.a	n.a	<0.001	0.23
Days to flower	Control	48	45	60	35	90				0.84
	Salt	48	46	60	36	95	<0.001	<0.001	0.26	0.84

*Measurements are on a per pot basis, with each pot consisting of two plants. Overall trait means, minimum and maximum values, and p-values for effects of genotype (G), treatment (T), and genotype by treatment interaction (G × T) are given for Genesis836, Rupali and a RIL population consisting of 200 genotypes. n.a is indicated for non-informative p-values where G × T was significant. Estimates of heritability (H^2^) for different traits under salt and control conditions is indicated*.

**Table 2:** Summary of measurements taken in 2017 Merredin field trial (Merredin2017) under salt and control conditions.

**Traits**	**Treatment**	**Genesis836**	**Rupali**	**Mean**	**Min**	**Max**	**G**	**T**	**G × T**	**Heritability** ** (H^2^)**
Seed yield (g)	Control	3.7	3.3	3.7	0.4	15.6				0.43
	Saline	5.2	3.7	4.3	0.4	17.1	<0.001	<0.001	0.965	0.35
Seed number	Control	19	15	19	2	68				0.66
	Saline	33	19	26	3	126	<0.001	<0.001	0.771	0.56
100-seed weight (g)	Control	19.2	22	20	4	58.8				0.71
	Saline	16.6	18.9	17.7	6.8	50.7	<0.001	<0.001	0.096	0.78
Number of total pods	Control	18	17	19	5	66				0.52
	Saline	29	19	24	4	100	<0.001	<0.001	0.739	0.45
Number of filled pods	Control	15	12	15	2	48				0.58
	Saline	24	15	19	2	90	<0.001	<0.001	0.831	0.49
Number of empty pods	Control	3	5	4	0	35				0.67
	Saline	4	4	5	0	26	<0.001	0.098	0.327	0.59
Harvest index	Control	0.43	0.31	0.44	0.04	3.09				0.31
	Saline	0.63	0.42	0.54	0.09	2.02	<0.001	<0.001	0.912	0.53
Plant height (cm)	Control	55.4	58.4	57.1	40	78				0.68
	Saline	55.2	53.8	53.5	26	82	<0.001	<0.001	0.998	0.64
Shoot biomass (g)	Control	9.4	11.1	9.3	1.7	21.4				0.49
	Saline	8.8	8.7	8.4	1.5	26	<0.001	<0.001	1	0.14
Potassium (μmol g^−1^ DW)	Control	652.2	611.5	657.6	389.4	1194.5				0.27
	Saline	1012.7	779.8	818.5	361.2	1806.2	0.555	0.555	0.943	0.07
Sodium (μmol g^−1^ DW)	Control	3.6	3.6	3.8	2.4	11.4				0.18
	Saline	5.4	5	5.1	3.1	11	<0.001	<0.001	0.979	0.41
Potassium: Sodium ratio	Control	209.5	193	199.7	76.1	1075.8				0.15
	Saline	196.8	167.6	178.8	49.5	492.2	<0.001	0.002	0.701	0.26
Chloride (μmol g^−1^ DW)	Control	20.2	24.5	19.7	11.9	28				0.31
	Saline	38.4	44.6	43.6	15.6	126.9	n.a	n.a	<0.001	0.26
Necrosis (1-9)	Control	1	1	1	1	1				n.a
	Saline	2	4	3	1	6	n.a	n.a	<0.001	0.63
Emergence	Control	14	13	13	1	18				0.44
	Saline	14	14	13	0	16	<0.001	0.21	0.832	0.54
Days to flower	Control	109	100	107	76	140				0.61
	Saline	110	97	107	78	144	<0.001	0.298	0.291	0.67
Days to podding	Control	131	123	129	110	152				0.46
	Saline	130	126	128	112	149	<0.001	0.004	0.267	0.47

*Overall trait means, minimum and maximum values, and p-values for effects of genotype (G), treatment (T), and genotype by treatment interaction (G × T) are given for Genesis836, Rupali and a RIL population consisting of 198 genotypes. n.a is indicated for non-informative p-values where G × T is significant. All measurements are made on per plant basis except emergence which is scored on per plot basis. Estimates of heritability (H^2^) for different traits under salt and control conditions is indicated*.

**Table 3:** Summary of measurements (per plant) taken in 2018 Merredin field trial (Merredin2018) under salt and control conditions.

**Trait**	**Treatment**	**Genesis836**	**Rupali**	**Mean**	**Min**	**Max**	**G**	**T**	**G × T**	**Heritability** ** (H^2^)**
Seed yield (g)	Control	2.9	2.7	3.3	2.0	4.8				0.14
	Salt	3.0	2.4	2.7	0.7	5.7	<0.001	<0.001	0.302	0.09
Harvest index	Control	0.46	0.43	0.43	0.33	0.54				0.61
	Salt	0.40	0.37	0.39	0.23	0.55	n.a	n.a	0.006	0.24
Shoot biomass (g)	Control	6.2	7.7	7.8	4.5	11.0				0.23
	Salt	6.7	6.4	7.3	2.9	12.9	<0.001	<0.001	0.418	0.09
Potassium (μmol g^−1^ DW)	Control	442.8	434.1	432.0	313.7	572.6				0.20
	Salt	388.6	352.4	373.4	248.3	576.8	<0.001	<0.001	0.528	0.14
Sodium (μmol g^−1^ DW)	Control	14.1	7.7	9.8	4.5	22.4				0.46
	Salt	80.2	48.6	60.3	9.5	221.0	n.a	n.a	<0.001	0.27
Potassium: Sodium ratio	Control	31.3	56.6	48.8	19.0	82.5				0.19
	Salt	4.9	7.3	8.2	1.7	28.5	n.a	n.a	0.025	0.20
Chloride (μmol g^−1^ DW)	Control	63.8	76.6	72.4	49.0	100.1				0.05
	Salt	380.0	401.3	365.2	244.9	535.1	0.6329	<0.001	0.062	0.19
Necrosis (1-9)	Control	1	1	1	1	1				n.a
	Salt	6	5	5	3	8	n.a	n.a	<0.001	0.24
Days to flower	Control	114	101	108	90	122				0.60
	Salt	119	110	113	96	126	<0.001	0.583	0.599	0.41
Days to pod	Control	129	119	124	111	134				0.53
	Salt	131	126	128	118	139	<0.001	<0.001	0.884	0.38

*Overall trait means, minimum and maximum values, and p-values for effects of genotype (G), treatment (T), and genotype by treatment interaction (G × T) are given for Genesis836, Rupali, and the RIL population consisting of 181 genotypes. n.a is indicated for non-informative p-values where G × T is significant. Estimates of heritability (H^2^) for different traits under salt and control conditions is indicated*.

The impact of salinity on the traits measured was dependent on the environment. Salinity negatively affected most of the traits measured in the glasshouse and Merredin2018 (Tables 1, 3) with the exception of harvest index in the glasshouse (Table 1). For instance, a seed yield reduction of 50, 34, and 16% was seen in the parents Rupali and Genesis836 and the RILs, respectively, under salinity in the glasshouse (Table 1). Similarly, a seed yield reduction of 11% and 18% was observed in Rupali and RILs in Merredin2018, respectively (Table 3). In contrast to our expectation, Genesis836 had 3% higher seed yield under salinity compared to control conditions (Table 3). Seed yield was not negatively impacted by salinity in Merredin2017 for either of the parents or the RILs. In contrast, seed yield under salinity was significantly higher (by 12, 44, and 16%) compared to the control treatment in Rupali, Genesis836, and RILs average, respectively, a similar observation made with other yield-related traits including number of pods, seed number and harvest index (Table 2). It is notable that seed yields and shoot biomass, were generally greater in Merredin2017 compared to Merredin2018, which was likely due to the greater water availability in 2017 with 73 mm of strategic irrigation events supplementing the 190 mm of growing season rainfall; that is, 263 mm of rainfall plus irrigation in 2017 compared with 216 mm of growing season rainfall only in 2018. In addition, the higher soil EC values and Na^+^ levels observed in the 2018 environment under both the control (higher background salinity) and saline (greater amount of NaCl applied in 2018 vs. 2017; see methods) conditions (Supplementary Figures 2, 3) possibly also interacting with the dry September in 2018, could also have contributed to the lower biomass and seed yields and lower heritability values in 2018 as compared with 2017.

Leaf necrosis was measured at early podding (~12 weeks) in field experiments. Necrosis levels (scores of proportion of whole shoot) were consistently three to five times higher in RILs and the parents under salinity treatment compared to controls in both years (Tables 2, 3). Necrosis distinguished the Rupali and Genesis836 parents in Merredin2017 and in the hydroponics experiment (Table 2, Supplementary Table 2), but not in Merredin2018 (Table 3). Salinity delayed flowering in Merredin2018 by 5 days (Table 3), a phenomenon that was not observed in the glasshouse nor in Merredin2017 (Tables 1, 2). However, G × T for days to flower was not significant for any of the three environments (Tables 1–3).

### Relationship Between Seed Yield and Yield-Related Traits

Seed yield under both control and saline conditions was positively and strongly correlated to seed number in the glasshouse (*r* = 0.78–0.80) and field (*r* = 0.84), a relationship which was highly significant (*p* ≤ 0.001) (Supplementary Tables 3, 4). Strong positive correlations of *r* = 0.78–0.84, *r* = 0.70–0.75, *r* = 0.78–0.87 under salt, and *r* = 0.80–0.84, *r* = 0.61–0.74, *r* = 0.82–0.84 under control were observed for seed yield with seed number, total pod number, and number of filled pods, respectively (Supplementary Tables 3, 4). This demonstrates the important role these traits play in yield determination under both salt and control conditions in both glasshouse and field conditions. Although seed size (100-seed weight) had a moderate positive correlation (*r* = 0.64) with seed yield under saline conditions in the glasshouse, it was not correlated with seed yield under saline conditions in the field or under control conditions in either environment (Supplementary Tables 3, 4). Shoot biomass had strong positive (*p* ≤ 0.001) correlations of *r* = 0.71 and *r* = 0.92 with seed yield under saline conditions in the glasshouse and Merredin2018, respectively, a relationship that was moderate (*r* = 0.48) in Merredin2017 (Supplementary Tables 3–5). Interestingly, the relationship of shoot biomass with seed yield under control conditions was environment-dependent. Shoot biomass had a negative, albeit weak correlation with seed yield (*r* = −0.33) in glasshouse conditions (Supplementary Table 3), perhaps due to a limitation of water and nutrient supply in the pot at seed filling stage. On the contrary, shoot biomass in the control treatment was positively correlated with seed yield in the two field environments, with correlation coefficients of *r* = 0.21 and *r* = 0.82 for Merredin2017 and Merredin2018, respectively (Supplementary Tables 4, 5). Plant height was strongly positively correlated with plant biomass in both the glasshouse (*r* = 0.90), and Merredin2017 (*r* = 0.65–0.68). The correlations between height and biomass closely reflected biomass/seed yield correlations in the glasshouse, while there was not a significant relationship between plant height and seed yield for Merredin2017. Plant heights were not recorded in Merredin2018.

Projected shoot area, measured at 7 weeks after sowing, was only weakly correlated with shoot biomass determined at maturity in the glasshouse under control (*r* = 0.07) and saline conditions (*r* = 0.10) (Supplementary Table 3), due to the indeterminate growth pattern of chickpea. Projected shoot area was most strongly correlated with water use, a trait also measured early during the growth period (*r* = 0.73–0.84). Both traits were more strongly correlated with seed yield under saline conditions (*r* = 0.27–0.30) than in the control treatment (*r* = 0.01–0.14) (Supplementary Table 3).

Leaf Na^+^ and Cl^−^ played a role in explaining biomass accumulation and seed yield in the different experimental environments. Na^+^ in the youngest fully expanded leaf was moderately negatively correlated (*r* = −0.49) with seed yield in the glasshouse under saline conditions (Supplementary Table 3). In contrast, this relationship was either very weak (*r* = 0.23) or non-significant (*r* = −0.14) in Merredin2017 and Merredin2018, respectively (Supplementary Tables 4, 5). Cl^−^ in the youngest fully expanded leaf was weakly correlated with seed yield under saline conditions in Merredin2017 (*r* = −0.23) and Merredin2018 (*r* = −0.35) (Supplementary Tables 4, 5). There was similarly a moderate negative relationship between Cl^−^ and shoot biomass in the field experiments (*r* = −0.30 in Merredin2017 and *r* = −0.36 in Merredin2018) (Supplementary Tables 4, 5). A strong positive relationship was observed between Cl^−^ and seedling biomass under hydroponics conditions (*r* = 0.80) (Supplementary Table 6), but this may be uninformative given weak correlations between biomass (projected shoot area) measured at early stages of growth and maturity, as seen in the glasshouse experiment (Supplementary Table 3).

**Figure 1:**
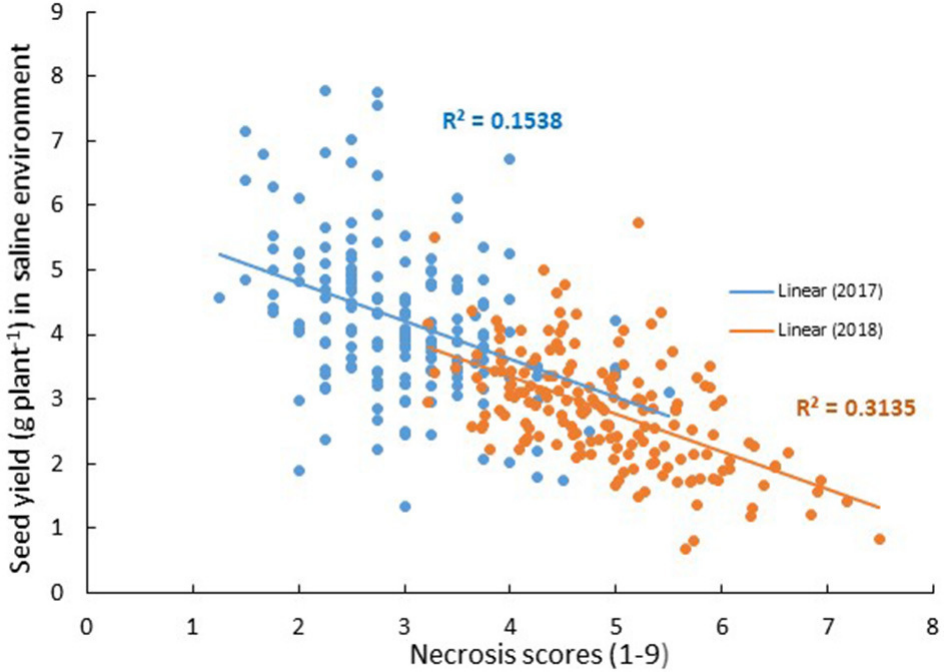
Relationship between seed yield under saline conditions and plant necrosis score (1–9, where 1 = no symptoms and 9 = dead) in Merredin2017 (blue) and Merredin2018 (orange) field trials. There was a modest negative relationship between seed yield under salinity and necrosis score across the two field experiments.

One of the easiest traits to assess is plant symptoms/necrosis. There was a modest negative linear relationship between leaf necrosis scores (as a proportion of the whole shoot) at early podding and seed yield under salinity across the two field experiments (Figure 1). Correlations of *r* = −0.4 and *r* = −0.56 between necrosis and seed yield under saline conditions in Merredin2017 and Merredin2018, respectively (Supplementary Tables 4, 5), indicated that necrosis scoring may be a suitable surrogate for selecting genotypes with high yield under salinity in field environments. There was a weak negative correlation between necrosis and biomass in the hydroponics experiment (*r* = −0.17) (Supplementary Table 6), but we were unable to determine if necrosis scores can predict yield in a controlled pot experiment. Necrosis scores in the hydroponics experiment did not correlate with scores obtained for each RIL in either field experiment (*r* = 0.16 in Merredin2017 and *r* = −0.03 in Merredin2018), suggesting that controlled-environment seedling screens may not reliably predict performance in the field.

### Impact of Flowering on Seed Yield

**Figure 2:**
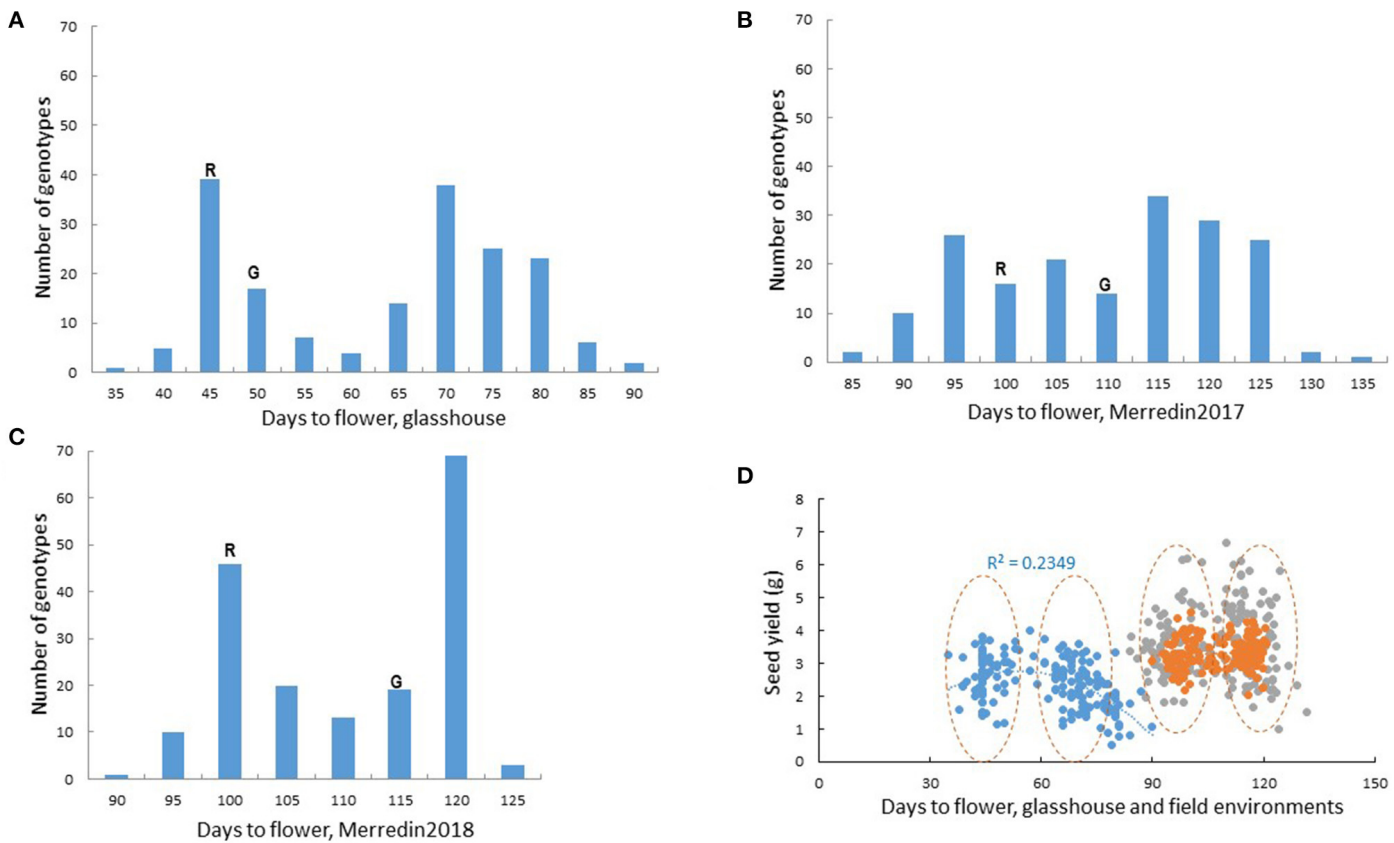
Flowering in the Rupali/Genesis836 RIL mapping population. Bimodal distribution for flowering time in the RIL population in **(A)** the glasshouse **(B)** Merredin2017 and **(C)** Merredin2018. Days to flower for the parents, Rupali(R) and Genesis836(G) is indicated. Transgressive segregation for flowering was evident in all the environments. **(D)** Influence of flowering on seed yield in the glasshouse and two field environments under non-saline conditions. Blue dots represent glasshouse data, gray dots represent data from Merredin2017 and orange dots represent data from Merredin2018. Generally, genotypes clustered into “early” and “late” groups as indicated by the dashed circles. Very early and very late flowering genotypes in the glasshouse had lower yields compared to the rest of the lines, a phenomenon that was not observed in the field.

Flowering and plant maturity are widely known to have a major, environment-dependent influence on seed yield. Days to flower in Rupali/Genesis836 followed a bimodal distribution both in the glasshouse and field experiements (Figure 2). Transgressive segregation was observed in all the experimental environments, with most RILs flowering much later compared to the parents in the glasshouse (Figure 2A). Genesis836 and Rupali time to flowering was equally progressively shortened by increasing daylength from 8 to 16 h in the growth room (Supplementary Figure 5). Similarly, extending photoperiod from 12 to 16 h drastically shortened days to flower, especially in the late flowering RILs (Supplementary Figure 5). In this study, flowering was seen to affect seed yield differently under glasshouse and field environments, with very early and very late lines having relatively lower yields compared to the rest of the lines in the glasshouse (Figure 2D). On the contrary, flowering did not have major impact on seed yield under either control or saline conditions in Merredin2017 (*r* = 0.02–0.09) or Merredin2018 (*r* = 0.08–0.23) (Figure 2D; Supplementary Tables 4, 5).

### Genetic Analysis

#### Rupali/Genesis836 Genetic Linkage Map

An intra-specific genetic map for Rupali/Genesis836 spanned 963.5 cM and consisted of 628 polymorphic markers mapped on 9 linkage groups (Table 4, Supplementary Figure 6). The number of markers and length of linkage groups varied, with linkage group (LG) 7, corresponding to CaLG07 of the Kabuli reference assembly v.1 (Varshney et al., 2013), having the most number of mapped markers (139). LG 1.2, corresponding to a partial length of CaLG01, had the least number of mapped markers (16) (Table 4). CaLG07 was densely populated with markers with an average spacing and maximum spacing between markers of 0.8 and 10.8 cM, respectively (Table 4). CaLG01 was split into two due to weak linkage resulting from an absence of markers to link the two sections (Table 4, Supplementary Figure 6). Of the nine linkage groups, CaLG04 had the longest genetic distance of 154.9 cM (Table 4, Supplementary Figure 6).

**Table 4:** Summary of the Rupali/Genesis836 genetic linkage map derived from 181 (F4:5) RIL mapping population.

**Linkage group**	**Chromosome number**	**Number of markers**	**Length (cM)**	**Average marker density (cM)**	**Maximum marker density (cM)**
L.1.1	CaLG01	33	71.9	2.2	18.9
L.1.2	CaLG01	16	2.8	0.2	1.7
L.2	CaLG02	109	143.5	1.3	16.3
L.3	CaLG03	49	121.5	2.5	20.1
L.4	CaLG04	126	154.9	1.2	12.2
L.5	CaLG05	48	143.5	3.1	14.3
L.6	CaLG06	59	138.4	2.4	28.0
L.7	CaLG07	139	111.3	0.8	10.8
L.8	CaLG08	49	75.7	1.6	12.4
Overall		628	963.5	1.6	28.0

*Genotyping was conducted by DArTseq and 628 high quality markers were clustered into nine linkage groups and optimally ordered using the MSTMap algorithm available in the ASMap package. Each linkage group has a corresponding chromosome number derived from anchoring the genetic map to the chickpea Kabuli reference assembly v.1. Length of each linkage group, average and maximum spacing between markers are indicated using centimorgan (cM) units. A visual illustration of the map can be found in Supplementary Figure 6*.

#### Flowering Loci Segragating in Rupali/Genesis836

**Figure 3:**
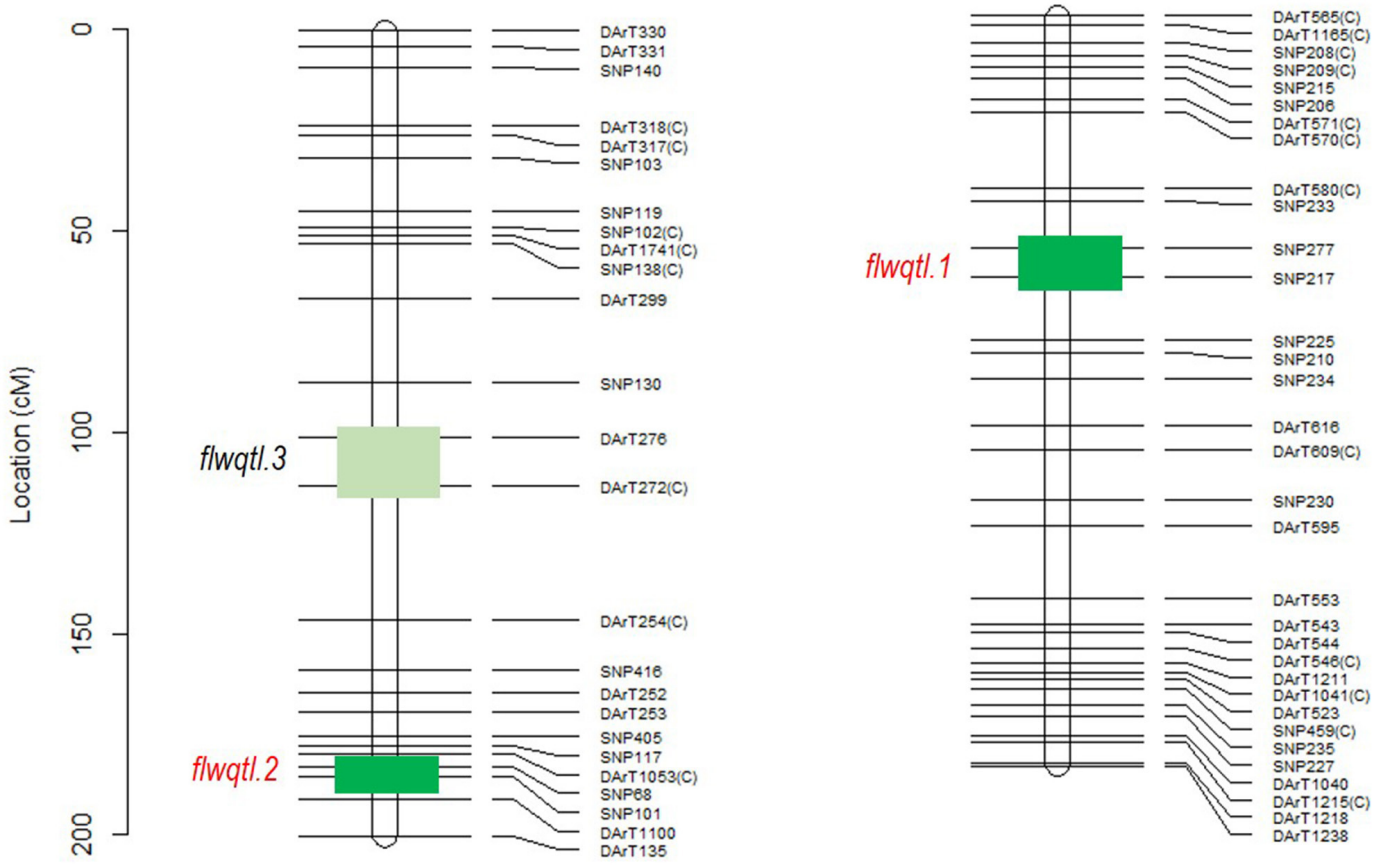
QTL analysis of flowering loci segregating in Rupali/Genesis836 population across three different environments; glasshouse, Merredin2017, and Merredin2018 under non-saline conditions. Green boxes show approximate genomic regions where the flowering loci map. Flowering QTL, *flwqtl.3* on CaLG03 was only identified in Merredin2018 while *flwqtl.1* and *flwqtl.2* on CaLG05 and CaLG03, respectively*, we*re identified in the glasshouse, Merredin2017 and Merredin2018 by both single environment QTL analysis and multi-environment QTL analysis. %GVE-percent genotypic variation explained.

Flowering time can have confounding effects on salinity tolerance. Therefore, we first investigated genetic control of flowering time in the Rupali/Genesis836 RIL population. Mapping for QTL controlling flowering in this population revealed three loci; two loci on CaLG03 and one on CaLG05 (Figure 3). The loci *flwqtl.1* (CaLG05) and *flwqtl.2* (CaLG03) were detected in the glasshouse and in Merredin2017 and Merredin2018 field experiments. In constrast, locus *flwqtl.3* on CaLG03 was only detected in Merredin2018 (Figure 3). Multi-environment QTL analysis utilizing BLUPs from the three different environments revealed two loci; locus *flwqtl.1* on CaLG05 with percentage genetic variation explained (GVE) of 81.1% and a LOD score of infinity (high-value allele from Genesis836), and locus *flwqtl.2* on CaLG03 (4.1% GVE) with a LOD score of 9.6 (high-value allele from Rupali) (Figure 3). We found that locus *flwqtl.1* corresponds to the *CaELF3a* gene reported in Ridge et al. (2017), and that Genesis836 and Rupali have contasting alleles for this gene, with Rupali carrying the early flowering mutated form, *caelf3a*.

#### QTL Mapped in Rupali/Genesis836

QTL analysis on the Rupali/Genesis836 population prior to adjusting for flowering found the majority of traits to locate to the same position as flowering loci on CaLG03 and CaLG05. After adjusting for flowering, 20 (under control conditions), 22 (under saline conditions), and 6 (salinity tolerance) significant QTL were identified (Tables 5, 6).

**Table 5:** Quantitative trait loci (QTL) associated with various traits measured across different environments including hydroponics, glasshouse and two field experiments (Merredin2017 and Merredin2018) under (A) saline and (B) control conditions QTL highlighted in blue and yellow represent major loci on CaLG04 controlling inherent growth-related traits and salinity specific traits, respectively.

**(A)**											
**Environment**	**Trait**	**QTL**	**Chromosome**	**Left marker**	**dist(cM)**	**Right marker**	**dist(cM)**	**Size**	**Prob**	**% Var**	**LOD**
Hydroponics	Seedling biomass	*salsBqtl.1*	CaLG04	SNP14_C14_12_74(C)	71.61	SNP15_C14_13_06	72.17	−0.1984	0	48.5	23.6345
Hydroponics	Seedling biomass	*salsBqtl.2*	CaLG01	SNP27	39.67	SNP23	50.16	0.1269	0	16.5	7.7398
Merredin2017	Chloride	*salClqtl.1*	CaLG05	DArT616	78.37	DArT610(C)	83.02	−0.0573	0	55.5	4.9976
Hydroponics	Chloride	*salClqtl.2*	CaLG04	SNP14_C14_12_74(C)	71.61	SNP15_C14_13_06	72.17	−2.828	0.0001	32.2	14.728
Merredin2017	Number of filled pods	*salFPqtl.1*	CaLG04	SNP201	27.87	SNP2_ Ca4_75	29.89	2.1991	0	21.3	6.9352
Merredin2018	Number of filled pods	*salFPqtl.2*	CaLG04	DArT417	88.95	SNP203	90.37	1.5338	0	15.1	5.1737
Merredin2018	Necrosis	*salNecrosisqtl.1*	CaLG05	DArT595	97.52	DArT553	111.41	−0.2285	1.00E−04	20.5	3.2408
Merredin2017	Necrosis	*salNecrosisqtl.2*	CaLG04	SNP148(C)	18.22	SNP201	27.87	−0.3491	0	39.1	12.6845
Merredin2018	Necrosis	*salNecrosisqtl.3*	CaLG04	SNP14_C14_12_74(C)	71.61	SNP15_C14_13_06	72.17	0.2697	0	34	5.4586
Hydroponics	Necrosis	*salNecrosisqtl.4*	CaLG03	DArT273(C)	68.63	DArT255(C)	88.75	−0.6299	0	41.9	12.8835
Merredin2017	Seed number	*salSNqtl.1*	CaLG04	SNP201	27.87	SNP2_ Ca4_75	29.89	3.6812	0	28.5	9.0112
Merredin2017	Seed number	*salSNqtl.2*	CaLG04	DArT417	88.95	SNP203	90.37	2.7537	0	23.2	7.5854
Merredin2017	Seed number	*salSNqtl.3*	CaLG01	DArT71	0	DArT1751	10.48	−1.9377	0	9.9	3.3289
Merredin2017	100–seed weight	*salSWqtl.1*	CaLG05	DArT595	97.52	DArT553	111.41	1.3526	0	21.8	20.5363
Glasshouse	100–seed weight	*salSWqtl.2*	CaLG04	SNP14_C14_12_74(C)	71.61	SNP15_C14_13_06	72.17	−1.5592	0	22.6	16.5896
Merredin2017	100–seed weight	*salSWqtl.3*	CaLG04	SNP14_C14_12_74(C)	71.61	SNP15_C14_13_06	72.17	−1.5393	0	34.4	22.3099
Merredin2017	Seed yield	*salSYqtl.1*	CaLG05	DArT595	97.52	DArT553	111.41	0.3222	0	17.9	4.3667
Merredin2017	Seed yield	*salSYqtl.2*	CaLG04	SNP201	27.87	SNP2_ Ca4_75	29.89	0.3865	0	22	5.1227
Glasshouse	Seed yield	*salSYqtl.3*	CaLG06	SNP246	131	SNP259(C)	133.94	0.3113	0	13.2	3.5456
Glasshouse	Water use efficiency	*salWUEqtl.1*	CaLG01	DArT85	27.79	DArT78(C)	28.07	484.6509	0	46.3	4.167
Glasshouse	X30AGR	*salX30AGRqtl.1*	CaLG01	SNP5	12.46	DArT1786	15.7	1.4136	0	28.7	4.0182
Glasshouse	X34AGR	*salX34AGRqtl.1*	CaLG01	SNP5	12.46	DArT1786	15.7	1.5533	0	29.2	3.735
**(B)**											
Glasshouse	Number of filled pods	*conFPqtl.1*	CaLG07	DArT1204(C)	43.73	SNP286(C)	46.27	−1.7159	0	19.6	3.7095
Merredin2017	Number of filled pods	*conFPqtl.2*	CaLG04	SNP14_C14_12_74(C)	71.61	SNP15_C14_13_06	72.17	1.8559	0	39.3	14.0499
Merredin2018	Sodium	*conNaqtl.1*	CaLG04	SNP201	27.87	SNP2_ Ca4_75	29.89	0.1007	0	17	4.3069
Glasshouse	Seed number	*conSNqtl.1*	CaLG07	DArT1046	40.32	DArT1204(C)	43.73	−2.5513	0	19.1	4.0558
Glasshouse	Seed number	*conSNqtl.2*	CaLG04	SNP14_C14_12_74(C)	71.61	SNP15_C14_13_06	72.17	2.1867	0	14.3	3.5923
Merredin2017	Seed number	*conSNqtl.3*	CaLG04	SNP14_C14_12_74(C)	71.61	SNP15_C14_13_06	72.17	2.1157	0	22.2	6.5191
Merredin2017	Seed number	*conSNqtl.4*	CaLG04	SNP202(C)	93.61	DArT1740(C)	103.27	1.7592	0	13.8	4.0847
Merredin2017	Seed number	*conSNqtl.5*	CaLG08	DArT1753	48.59	SNP393(C)	55.61	1.2955	0	8.5	3.776
Merredin2017	100-seed weight	*conSWqtl.1*	CaLG05	DArT595	97.52	DArT553	111.41	1.411	0	17.5	13.2902
Glasshouse	100-seed weight	*conSWqtl.2*	CaLG04	SNP14_C14_12_74(C)	71.61	SNP15_C14_13_06	72.17	−1.9649	0	35.2	23.9314
Merredin2017	100–seed weight	*conSWqtl.3*	CaLG04	SNP14_C14_12_74(C)	71.61	SNP15_C14_13_06	72.17	−1.9341	0	39.7	31.4225
Glasshouse	100-seed weight	*conSWqtl.4*	CaLG06	SNP246	131	SNP259(C)	133.94	0.9269	0	8.3	5.5955
Merredin2018	Seed yield	*conSYqtl.1*	CaLG05	DArT523	126.94	DArT1040	137.75	−0.1348	0	39.5	4.1562
Merredin2017	Seed yield	*conSYqtl.2*	CaLG04	DArT419	88.67	DArT417	88.95	0.2063	0.0001	11.3	3.0544
Merredin2017	Seed yield	*conSYqtl.3*	CaLG01	DArT63(C)	24.06	SNP24(C)	26.68	−0.2385	0	14.2	4.0241
Glasshouse	Water use	*conWUqtl.1*	CaLG04	SNP14_C14_12_74(C)	71.61	SNP15_C14_13_06	72.17	−2.5546	1.00E-04	22.1	3.0077
Glasshouse	Water use efficiency	*conWUEqtl.1*	CaLG04	SNP14_C14_12_74(C)	71.61	SNP15_C14_13_06	72.17	−2.71E+02	0	18.7	4.0655
Glasshouse	Water use efficiency	*conWUEqtl.2*	CaLG01	DArT1786	15.7	DArT1798	15.97	284.3119	0	21	4.6183
Glasshouse	X30AGR	*conX30AGRqtl.1*	CaLG04	DArT417	88.95	SNP203	90.37	−2.6217	0	64.1	7.6002
Glasshouse	X34AGR	*conX34AGRqtl.1*	CaLG04	DArT417	88.95	SNP203	90.37	−3.0783	0	64	7.8203

*X30AGR-Absolute growth rate at 30 days after sowing, X34AGR- Absolute growth rate at 34 days after sowing. Allele effects contributed by Rupali are denoted by negative effect size while the positive effect size is contributed by Genesis836*.

**Table 6:** Salinity tolerance QTL: QTL obtained from residuals from the regression line when salinity BLUPs are regressed on control BLUPs.

**Environment**	**Trait**	**QTL**	**Chromosome**	**Left Marker**	**dist(cM)**	**Right Marker**	**dist(cM)**	**Size**	**Prob**	**% Var**	**LOD**
Merredin2017	Number of filled pods	*saltolFPqtl.1*	CaLG04	SNP201	27.87	SNP2_ Ca4_75	29.89	0.0321	0.0001	7.3	3.1013
Merredin2017	Seed number	*saltolSNqtl.1*	CaLG04	SNP201	27.87	SNP2_ Ca4_75	29.89	0.0338	0	13	5.616
Merredin2017	Seed number	*saltolSNqtl.2*	CaLG06	SNP262	118.47	SNP278	127.29	0.0216	0	7.1	3.3706
Merredin2017	Seed yield	*saltolSYqtl.1*	CaLG05	DArT595	97.52	DArT553	111.41	0.0043	0	8.5	4.4936
Merredin2017	Seed yield	*saltolSYqtl.2*	CaLG04	SNP201	27.87	SNP2_ Ca4_75	29.89	0.0049	0	9.6	4.9432
Glasshouse	Water use	*saltolWUqtl.1*	CaLG04	SNP201	27.87	SNP2_ Ca4_75	29.89	−0.0164	1.00E-04	8.8	3.2673

*Merredin2017−2017 Merredin salinity field experiment. The QTL highlighted in yellow locate to a common region on CaLG04 observed to control a majority of salinity tolerance related traits. Allele effects contributed by Rupali are denoted by negative effect size while the positive effect size is from Genesis836*.

**Figure 4:**
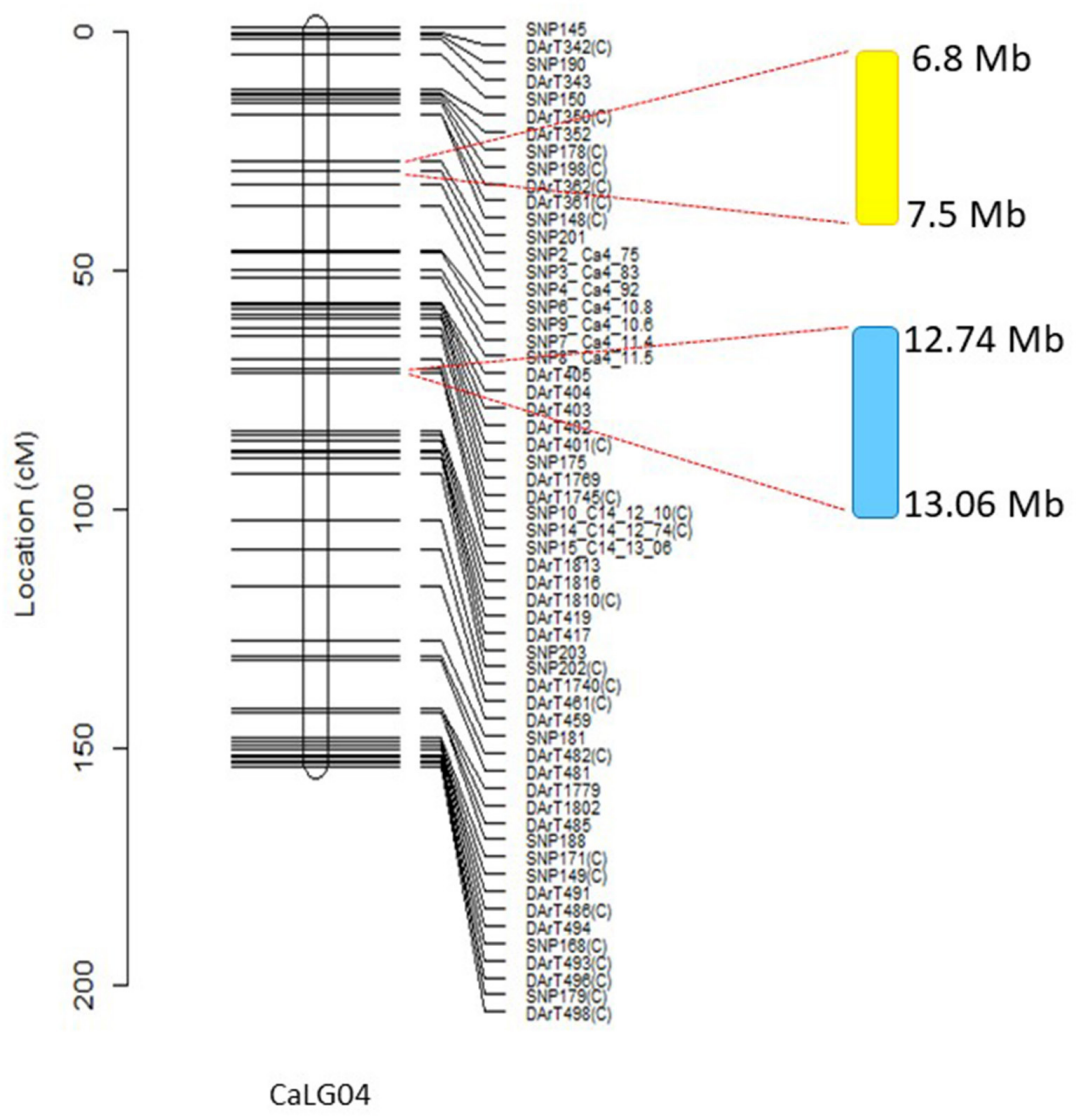
Major salinity and seed yield or biomass-related QTL on CaLG04. Genomic region highlighted in yellow shows the location of multiple salinity-specific QTL associated with necrosis scores and salinity tolerance *per se*, calculated from residuals from regressions of traits measured in salinity onto corresponding control values. These traits included number of filled pods, seed number, and seed yield. Genomic region highlighted in blue relates to the position of QTL for traits controlling 100-seed weight, above ground biomass, and seed number in both control and salt treatments across different environments. Physical positions of molecular markers flanking the QTLs are provided using the Kabuli reference assembly v.2.

It was not uncommon to observe genomic regions that only appear under control conditions and not saline conditions and vice-versa. For example, *conFPqtl.1* and *conSNqtl.1* on CaLG07 controlled number of filled pods and seed number, respectively, under control but not saline conditions (Table 5). Regions that only appeared under saline conditions and not control conditions included *salsBqtl.2, salClqtl.1, salFPqtl.1, salNecrosisqtl.2, salSNqtl.1*, and *salSYqtl.2* (Table 5). Similarly, certain loci were environment-specific. For instance, a locus on CaLG07 (*conFPqtl.1* and *conSNqtl.1*) controlled number of filled pods (19.6% GVE) and seed number (19.1% GVE) under control conditions in the glasshouse but not in Merredin2017 or Merredin2018. *conSNqtl.4* only controlled seed number (13.8% GVE) under control conditions in Merredin2017 (Table 5). Interestingly, many loci were seen to control traits under both control and saline conditions, suggesting their role in plant growth rather than salt tolerance *per se*. For instance, a locus on CaLG04 with a genetic distance of 0.56 cM was seen to control number of filled pods, seed number, 100-seed weight, water use, and water use efficiency (18.7−39.7% GVE) under control conditions in multiple environments (Figure 4, Table 5). Likewise, the same locus controlled seedling biomass, Cl^−^, necrosis, and 100-seed weight (22.6–48.5% GVE) under saline conditions (Figure 4, Table 5). Other plant growth and yield-related QTL include *conSYqtl.2* and *salSNqtl.2* on CaLG04, controlling seed yield under control conditions (11.3% GVE) and seed number under saline conditions (23.2% GVE), respectively, in Merredin2017 (Table 5). A region on CaLG05 controlled seed yield (*salSYqtl.1*) and necrosis *(salNecrosisqtl.1*) under saline conditions (17.9% GVE and 20.5% GVE, respectively), and 100-seed weight (*salSWqtl.1* and *conSWqtl.1*) under saline (21.8% GVE) and control conditions (17.5% GVE), respectively (Table 5).

**Figure 5:**
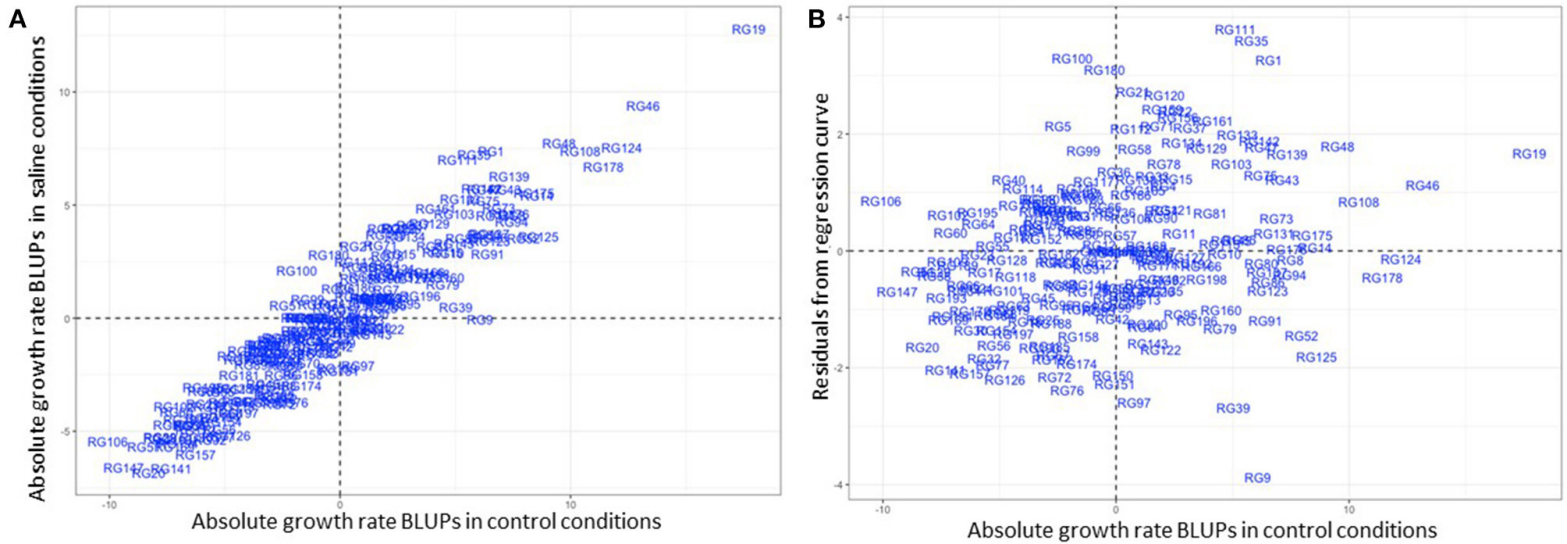
An example of a salinity tolerance measure: Regression of salinity treatment-derived best linear unbiased predictors (BLUPs) on control treatment-derived BLUPs, with the residuals used to quantify salinity tolerance. This example shows absolute growth rate (AGR) obtained in the glasshouse, with the graph on the left showing salinity treatment-derived AGR BLUPs plotted against control treatment-derived BLUPs. The graph on the right shows residuals from the regression curve plotted against control treatment-derived BLUPs. A random distribution of the data shows there was no influence of AGR in control conditions on the residuals. Genotypes in the two upper quadrants demonstrate salinity tolerance with respect to this trait.

A closer observation of CaLG04 revealed two distinct clusters of QTL (Table 5, Figure 4). QTL for yield-related traits observed only in saline treatments (number of filled pods, seed number and seed yield), and for necrosis measured under saline conditions, mapped at a physical location of 6.8 Mb-7.5 Mb based on the Kabuli reference assembly v.2 (Figure 4). Inherent growth/yield-related traits (100-seed weight, seedling biomass and plant biomass in both control and saline conditions) mapped at 12.74–13.06 Mb. To identify QTL for salinity tolerance *per se*, salinity BLUPs for each trait were regressed onto control BLUPs and the residuals from these regressions used in QTL analysis. An example using absolute growth rate (AGR) from the glasshouse experiment is illustrated in Figure 5. Using this residuals analysis method, three genomic regions (on CaLG04, CaLG05, CaLG06) were found to control salinity tolerance *per se* in Rupali/Genesis836 (Table 6). The locus on CaLG04 (7.3–13% GVE) with a LOD score of 3.1–5.6 controlled number of filled pods (*saltolFPqtl.1*), seed number (*saltolSNqtl.1*), and seed yield (*saltolSYqtl.1*) in Merredin2017, with Genesis836 contributing the high-value allele (Table 6). Additionally, the same region controlled salinity tolerance water use (*saltolWUqtl.1*) (8.8% GVE, LOD score 3.3) in the glasshouse, with the high-value allele contribution from Rupali (Table 6). Salinity tolerance seed number from Merredin2017 was controlled by *saltolSNqtl.2* on CaLG06 (7.1% GVE, LOD score 3.4), with the high-value allele contribution from Genesis836 (Table 6). Salinity tolerance seed yield from Merredin2017 was controlled by *saltolSYqtl.1* on CaLG05 (8.5% GVE, LOD score 4.5), with the high-value allele contribution from Genesis836 (Table 6).

## Discussion

This study reports the genetic basis of salinity tolerance in chickpea through extensive high-precision phenotyping of a Rupali/Genesis836 RIL population, developed through aSSD, at different developmental stages in different environments whilst controlling for flowering time. The findings are supported by hydroponic and soil-based assays in the glasshouse and soil based assays in the field. Phenotypic data were combined with a genetic map to identify important genome segments, with relevance to Australian environments, that control salinity tolerance. This information will be used by the breeding program to improve the accuracy and rate of genetic gain in developing more salt tolerant chickpea varieties.

Salinity tolerance is a complex trait which is widely considered in literature to be polygenic, quantitative and highly influenced by the environment. Indeed, our study highlights the importance of phenotyping for salinity tolerance under different environments prior to making selection decisions on the salinity tolerance status of different genotypes. The growing environment played a large role in the expression of salinity tolerance. For example, necrosis scores obtained in the field environments correlated poorly with those obtained in hydroponics. We also observed considerable differences between the two field experiments, with seed yield and shoot biomass greater in Merredin2017 compared to Merredin2018, and Na^+^ content in the youngest fully expanded leaf at early podding of plants in saline conditions averaging 12-fold higher in the Merredin2018 experiment compared to Merredin2017 (Tables 2, 3). Seed yield was not reduced by salinity in the Merredin2017 experiment (Table 2). Consequently, the severity of the imposed salinity treatment was increased for the Merredin2018 experiment. Despite this, necrosis correlated well with seed yield under salinity in both field experiments, and genetic analysis of the Merredin2017 experiment indicated these data more closely aligned with high-throughput image-based phenotyping in the glasshouse (Table 5), and revealed a greater number of saline treatment-specific and salinity tolerance loci (Tables 5, 6; discussed below) compared to Merredin2018. Given the magnitude of the differences between phenotyping experiments, also indicated by differences in heritability values for traits common between the different environments, a multi-environment genetic analysis approach was not considered to be warranted.

Flowering plays a major role in influencing crop duration and thus crop adaptation to environment. It is therefore important to eliminate, where possible, the confounding effects of flowering time by selecting genotypes with similar phenology as parents for mapping population development (Pinto et al., 2010). Rupali and Genesis836 were chosen as parents because, aside from contrasting for salinity tolerance, they displayed a restricted range in flowering time. However, transgressive segregation, likely due to complementary gene action, was observed for days to flowering in the RIL population. Increasing day length reduced the observed flowering time gap in the RILs, both during aSSD generation cycling and when phenotyped under controlled conditions (Supplementary Figure 5). However, phenotyping plants under artificial long-day conditions would have an impact on plant development and may not be optimal for studying salinity tolerance and/or yield-related traits. We identified two major flowering loci (*flwqtl.1* on CaLG05 and *flwqtl.2* on CaLG03) segregating in the Rupali/Genesis836 population (Figure 3). The QTL *flwqtl.1* on CaLG05, driving most of the phenotypic variation for flowering, corresponds to the *Efl1* locus for which the underlying gene is *CaEFL3a*, an ortholog of Arabidopsis EARLY FLOWERING3 (ELF3) (Ridge et al., 2017). Rupali carries the early flowering variant of this gene. In contrast, the Genesis836 allele for *flwqtl.2* reduced days to flower (Figure 3). *flwqtl.1* and *flwqtl.2* were incorporated as co-factors during subsequent genetic analysis of Rupali/Genesis836, to ensure the identification of true salinity tolerance regions not confounded by flowering or maturity. A minor QTL for flowering, *flwqtl.3*, was observed on CaLG03 in a single environment (Merredin2018; Figure 3), with the region corresponding to a cluster of FT genes reported to control time to flowering and growth habit in several chickpea populations (Ortega et al., 2019). However, time to flower could not be consistently attributed to this locus in the Rupali/Genesis836 population, and it was therefore not included as an additional co-factor.

We found that leaf necrosis scored at early podding in the field may be a suitable surrogate for salinity tolerance in chickpea. A QTL for necrosis in Merredin2017 (*salNecrosisqtl.2*; Table 5), on CaLG04, corresponded to a salinity tolerance *per se* region consistently identified for a number of traits in both Merredin2017 and from the glasshouse experiment using a residuals analysis method (Table 6 and discussed below). Additionally, necrosis scoring from both Merredin2017 and Merredin2018 was moderately correlated with the yield of genotypes in the high-saline treatment (*R*^2^ = 0.15–0.31; Figure 1). Previously, Maliro et al. (2008) proposed selection of salinity-tolerant chickpea lines (landraces and wild relatives) using necrosis scores and biomass cuts of 6-week-old seedlings grown in sand and gravel irrigated with nutrient solution with added salinity. This methodology is currently utilized in the Australian breeding program to select for salt tolerant genotypes. Here, we tried to replicate this set-up to screen the Rupali/Genesis836 population, to investigate how such a system compares with soil-based experiments in the glasshouse and in the field. Unfortunately, the hydroponics set-up did not compare well with the other environments. Genotypes ranked differently for necrosis in the two phenotyping systems, and genetic analysis using necrosis data from the hydroponics experiment did not reveal any salinity specific QTL in common with the other environments. The QTL *salNecrosisqtl.4* on CaLG03 (Table 5) was not identified for any other trait in any environment, although it did co-locate with the minor flowering region, *flwqtl.3* (Figure 3). This finding emphasizes the need to screen plants in different environments, and suggests it may be necessary to employ screens that extend beyond the seedling stage in order to make accurate inferences on the tolerance status of plants. Whilst we acknowledge that necrosis at podding/reproductive growth stage is not an early indicator of salinity tolerance, the use of this trait as a proxy for tolerance may circumvent the need to compare performance of plants in high- and low-saline treatments.

Several studies in chickpea have used relative measures between salt and control treatments and developed salinity tolerance indices to define salinity tolerance, as opposed to only looking at performance under stress (Vadez et al., 2007; Soren et al., 2020). In an approach similar to Vadez et al. (2007), we determined salinity tolerance *per se* by comparing residuals derived from regressing BLUPs for each trait from a salinity treatment onto BLUPs from the control treatment. Genetic analyses utilized absolute measurements from control treatments and absolute measurements from salinity treatments, as well as the residuals derived from regressions as described. We identified genomic regions that were unique to either control or salinity treatments, as well as regions common to both treatments. We also identified loci in common with previous salinity studies in chickpea. For example, a genomic region on CaLG07 (13.6 Mb- 37.5 Mb Kabuli reference assembly v.1), regulating both number of filled pods (*conFPqtl.1*) and seed number (*conSNqtl.1*) in the control treatment in the glasshouse, was also identified by Vadez et al. (2012a) and Pushpavalli et al. (2015a) linked to above-ground dry matter, total pod number, seed number and harvest index, under saline treatments.

Many loci identified in this study were found under both salt and control conditions, implying their role in the general regulation of plant growth and yield rather than to salinity tolerance *per se*, a phenomenon that has also been observed in bread wheat (Genc et al., 2019), wild barley (Saade et al., 2016) and recently in sunflower (Temme et al., 2020). For instance, a region on CaLG04 was found to control plant biomass, Cl^−^ content, leaf necrosis scores and 100-seed weight (22.6–48.5% GVE; Table 5) under salinity treatment, and number of filled pods, seed number, 100-seed weight, water use, and water use efficiency (14.3–39.3% GVE) under control treatment. Previously referred to as the “QTL hotspot,” this locus has been reported to be associated with drought tolerance (Kale et al., 2015) and plant vigor (Sivasakthi et al., 2018). Further analysis of the CaLG04 region in Australian growing environments and adapted germplasm is warranted to determine its role in maintaining yield and yield stability.

Four loci were identified that were unique to salinity treatments (Table 5). These include three distinct regions controlling seedling biomass (*salsBqtl.2*) and water use efficiency (*salWUEqtl.1*) on CaLG01, Cl^−^ content (*salClqtl.1*) on CaLG05, and a region on CaLG04 containing a cluster of QTL controlling number of filled pods (*salFPqtl.1*), leaf necrosis (*salNecrosisqtl.2*), seed number (*salSNqtl.1*), and seed yield (*salSY.2*). The region on CaLG04 was distinct from the CaLG04 location harboring QTL controlling traits common to both control and salinity treatments. This region is estimated to be 5 Mb proximal to the “QTL hotspot” (Kabuli reference assembly v.2). Genetic analysis of salinity tolerance *per se*, using the residuals method, identified the same salinity-specific region on CaLG04, containing several QTL relating to water use (*saltolWUqtl.1*), number of filled pods (*saltolFPqtl.1*), seed number (*saltolSNqtl.1*), and seed yield (*saltolSYqtl.2*), with the high-value allele contribution from the salt-tolerant Genesis836 parent (7.3–13% GVE; Table 6). Clearly, this demonstrates the role of the CaLG04 genomic region in controlling salinity tolerance in chickpea. Using the residuals method, two other regions, *saltolSNqtl.2* (CaLG06) and *saltolSYqtl.1* (CaLG05), were identified to associate with salinity tolerance in this study (Table 6). The region on CaLG05 also harbored QTL controlling leaf necrosis and seed yield under salinity in the field, as well as 100-seed weight under both control and salinity treatments (Table 5).

In conclusion, data were obtained from high-throughput phenotyping in a controlled environment and from field phenotyping across 2 years, for a bi-parental chickpea RIL population developed using parents selected for their contrasting salinity tolerance. We have utilized those data in genetic analyses controlling for flowering time and inherent growth differences, to decouple plant growth from salinity tolerance, and identified two distinct genomic regions on CaLG04. The development and validation of molecular markers closely linked to regions specific for salinity tolerance on CaLG04, CaLG05 and CaLG06, as well as the growth/yield-related region on CaLG04, should enable selection of both sets of genomic regions and/or traits associated with these regions for future germplasm improvement in the Australian chickpea breeding program.

## Data Availability Statement

The datasets presented in this study can be found in online repositories. The names of the repository/repositories and accession number(s) can be found in the article/Supplementary Material.

## Author Contributions

JA, TDC, JH, YL, PL, and TS conceived and designed the experiments. JA initiated RIL population development. JC progressed RIL generations using accelerated-Single Seed Descent (aSSD). JA, TDC, DTN, DN, JQ, LK, and JH performed the experiments and generated the data. CB assisted with experimental design for glasshouse experiments. JA, CB, and JT analyzed the data. JA wrote the manuscript, with significant contributions from TDC, JT, JC, JH, YL, and TS. All authors assisted in interpretation of data and manuscript proof reading and have read and agreed to the published version of the manuscript.

## Conflict of Interest

The authors declare that the research was conducted in the absence of any commercial or financial relationships that could be construed as a potential conflict of interest.
