# Supplementary material for: Novel Salinity Tolerance Loci in Chickpea Identified in Glasshouse and Field Environments
Source: Front Plant Sci. 2021 Apr 28;12:667910. doi: 10.3389/fpls.2021.667910 (PMC8113763; doi:10.3389/fpls.2021.667910)
Supplement: Supplementary file 1 [file Presentation_1.pptx]

## Slide 1
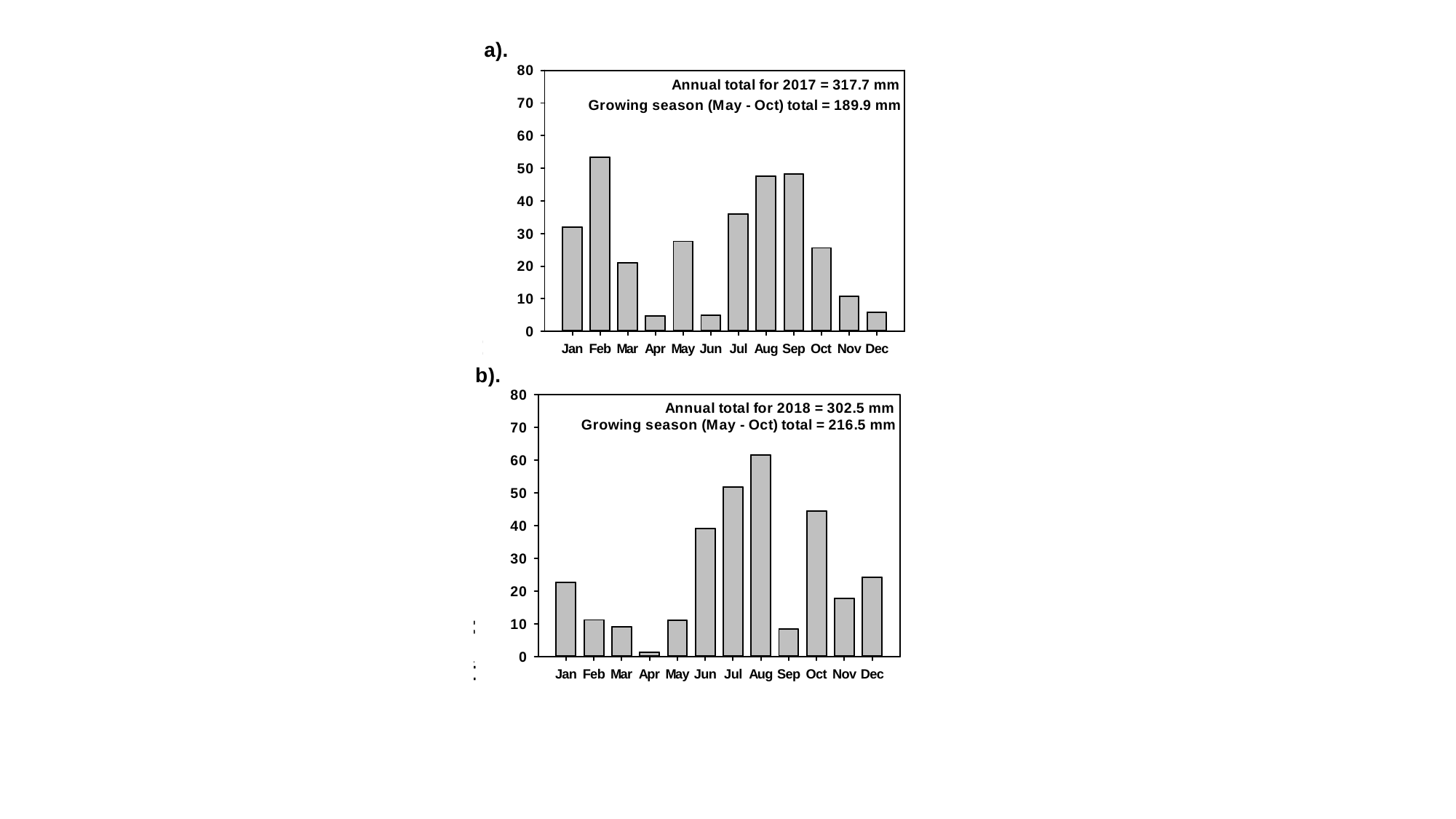

## Slide 2
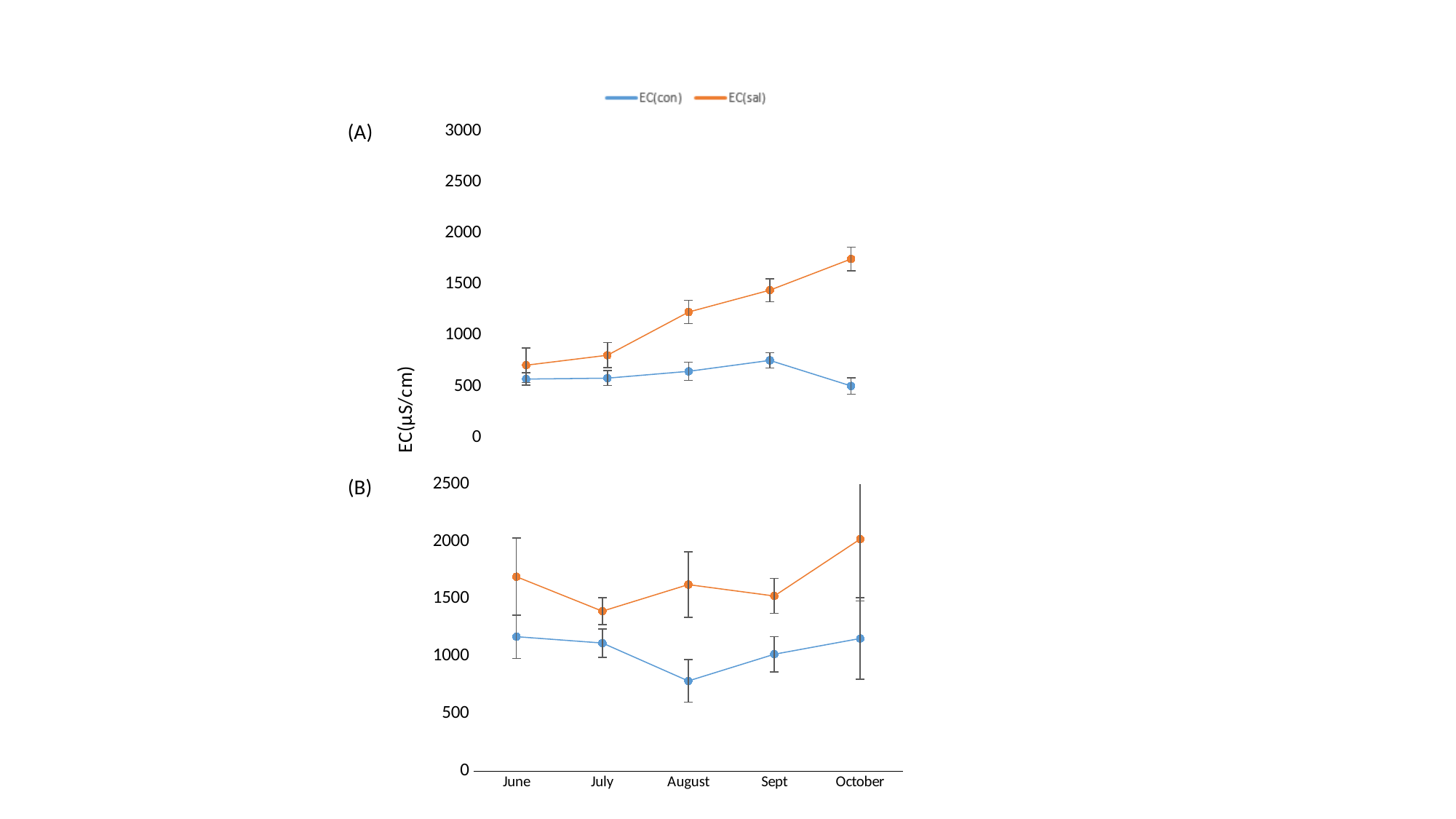

(A)
### Chart
| Category | EC(con) | EC(sal) |
|---|---|---|
| June | 572.2 | 707.8 |
| July | 581.1 | 805.4 |
| August | 647.7 | 1228.7 |
| September | 754.7 | 1442.5 |
| October | 505.0 | 1747.6 |(B)
### Chart
| Category | EC-Av con | EC-Av sal |
|---|---|---|
| June | 1170.3333333333333 | 1692.6666666666667 |
| July | 1115.0 | 1392.6666666666667 |
| August | 785.0 | 1623.6666666666667 |
| Sept | 1017.3333333333334 | 1524.6666666666667 |
| October | 1154.6666666666667 | 2020.0 |

## Slide 3
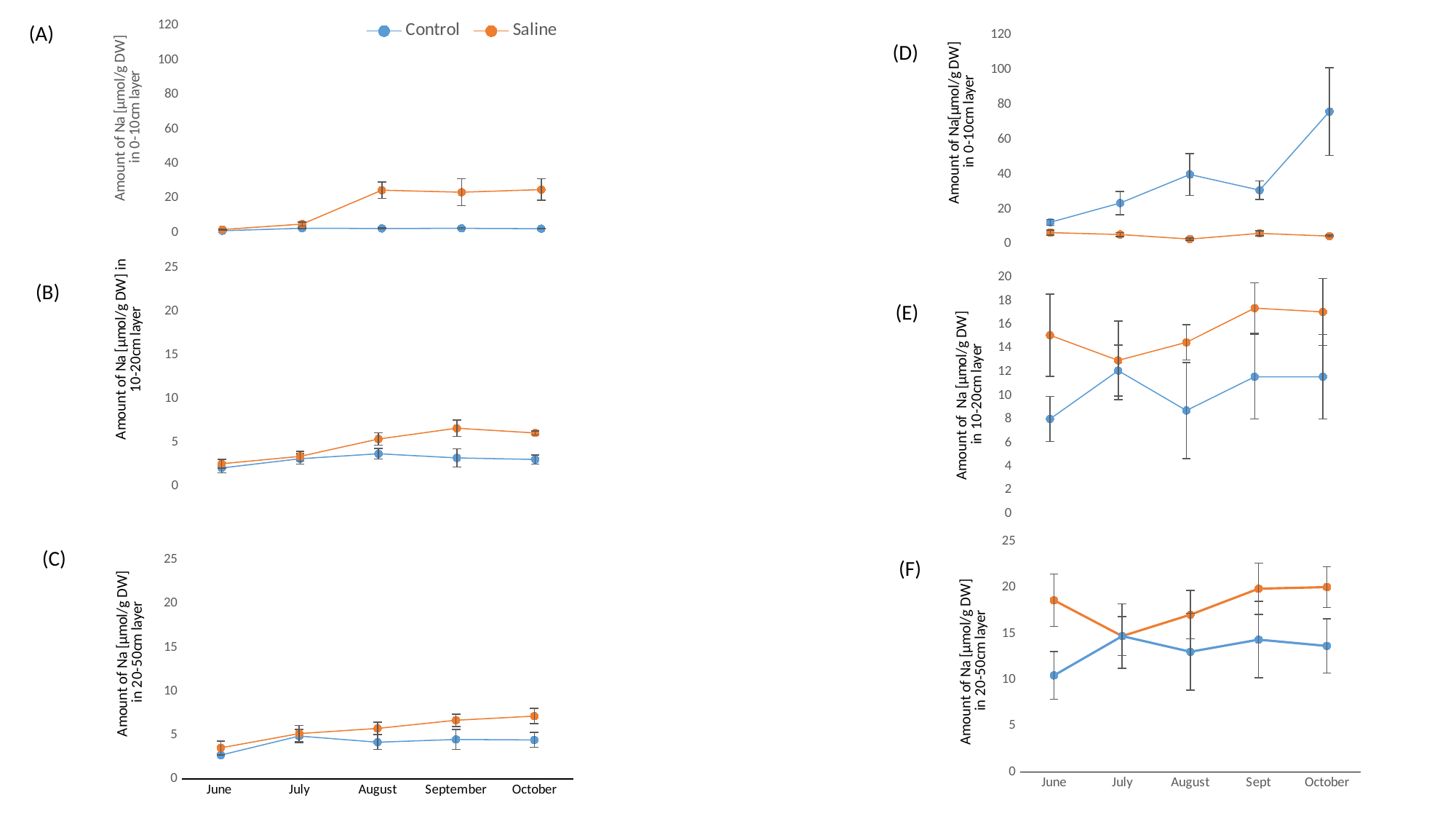

### Chart
| Category | Control | Saline |
|---|---|---|
| June | 1.156920400173989 | 1.8522488038277514 |
| July | 2.5989865158764687 | 5.0242279251848645 |
| August | 2.4589734667246637 | 24.61909525880818 |
| September | 2.5487690300130494 | 23.456120052196614 |
| October | 2.3379295345802524 | 24.97763157894737 |(A)
### Chart
| Category | Sal-Na av | Con-Na av |
|---|---|---|
| June | 12.53481 | 6.606594 |
| July | 23.5619 | 5.518341 |
| August | 39.93441 | 2.909091 |
| Sept | 30.95603 | 6.1893 |
| October | 76.07127 | 4.601966 |(D)
### Chart
| Category | Control | Saline |
|---|---|---|
| June | 2.103140495867769 | 2.583418877772945 |
| July | 3.161505002174859 | 3.4320835145715534 |
| August | 3.730291431056982 | 5.415859069160506 |
| September | 3.2560330578512398 | 6.650900391474553 |
| October | 3.07281426707264 | 6.107177033492824 |
### Chart
| Category | Sal-Na av | Con-Na av |
|---|---|---|
| June | 15.10017 | 8.016703 |
| July | 12.97416 | 12.10938 |
| August | 14.50109 | 8.733362 |
| Sept | 17.39226 | 11.5897 |
| October | 17.07217 | 11.5897 |(B)
(E)
### Chart
| Category | Sal-Na av | Con-Na av |
|---|---|---|
| June | 18.61105 | 10.46281 |
| July | 14.7271 | 14.7271 |
| August | 17.04567 | 13.03088 |
| Sept | 19.86265 | 14.34735 |
| October | 20.03588 | 13.661 |(C)
### Chart
| Category | Control | Saline |
|---|---|---|
| June | 2.733953892996955 | 3.5583123096998697 |
| July | 4.904602000869945 | 5.187999130056547 |
| August | 4.201678990865594 | 5.772970856894303 |
| September | 4.514963027403219 | 6.707481513701609 |
| October | 4.459612875163114 | 7.173945193562419 |(F)

## Slide 4
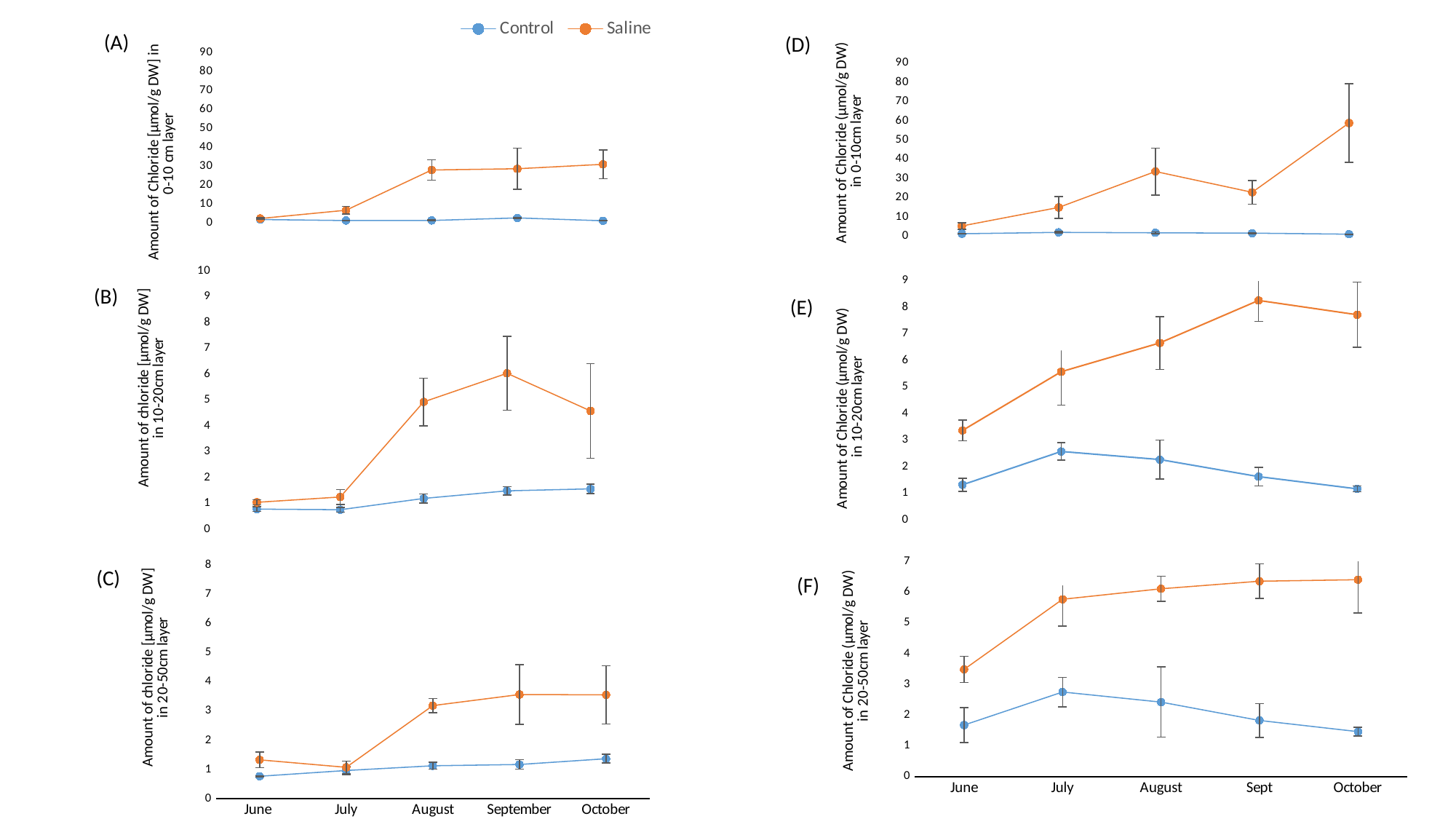

### Chart
| Category | Sal-Cl av | Con-Cl av |
|---|---|---|
| June | 5.325337 | 1.293477 |
| July | 14.97413 | 2.004307 |
| August | 33.6737 | 1.787041 |
| Sept | 22.8483 | 1.599869 |
| October | 58.93427 | 1.051928 |
### Chart
| Category | Control | Saline |
|---|---|---|
| June | 1.8729472916666667 | 2.4234801041666674 |
| July | 1.3298427499999996 | 6.783013749999999 |
| August | 1.3760238 | 28.04492725 |
| September | 2.7243276 | 28.700432 |
| October | 1.2144626666666667 | 31.0504675 |(A)
(D)
### Chart
| Category | Control | Saline |
|---|---|---|
| June | 0.7848854166666668 | 1.046355703125 |
| July | 0.7608110625 | 1.2539992083333331 |
| August | 1.1973296000000002 | 4.93336075 |
| September | 1.491041825 | 6.041047000000001 |
| October | 1.5675498333333333 | 4.582668 |
### Chart
| Category | Sal-Cl av | Con-Cl av |
|---|---|---|
| June | 3.371512 | 1.328023 |
| July | 5.582298 | 2.581501 |
| August | 6.66519 | 2.277682 |
| Sept | 8.262749 | 1.634897 |
| October | 7.725398 | 1.175235 |(B)
(E)
### Chart
| Category | Sal-Cl av | Con-Cl av |
|---|---|---|
| June | 3.491897 | 1.678026 |
| July | 5.774095 | 2.755359 |
| August | 6.116253 | 2.426157 |
| Sept | 6.36217 | 1.828052 |
| October | 6.412123 | 1.463955 |
### Chart
| Category | Control | Saline |
|---|---|---|
| June | 0.769714375 | 1.33162546875 |
| July | 0.9665418750000001 | 1.0752320833333333 |
| August | 1.1280605000000001 | 3.1890845 |
| September | 1.171893275 | 3.56714735 |
| October | 1.3693628333333334 | 3.5569560000000005 |(C)
(F)

## Slide 5
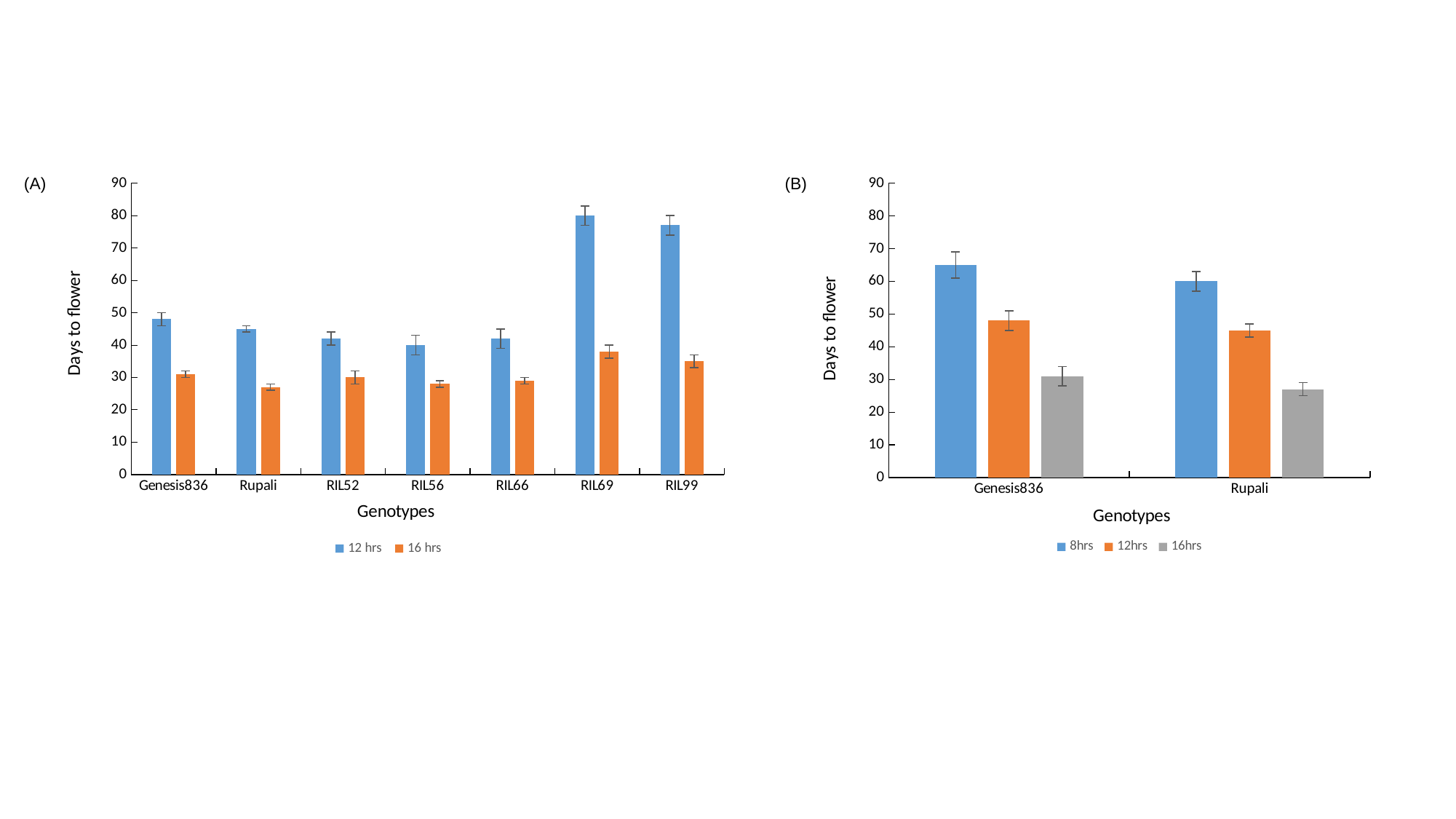

(A)
### Chart
| Category | 12 hrs | 16 hrs |
|---|---|---|
| Genesis836 | 48.0 | 31.0 |
| Rupali | 45.0 | 27.0 |
| RIL52 | 42.0 | 30.0 |
| RIL56 | 40.0 | 28.0 |
| RIL66 | 42.0 | 29.0 |
| RIL69 | 80.0 | 38.0 |
| RIL99 | 77.0 | 35.0 |(B)
### Chart
| Category | 8hrs | 12hrs | 16hrs |
|---|---|---|---|
| Genesis836 | 65.0 | 48.0 | 31.0 |
| Rupali | 60.0 | 45.0 | 27.0 |

## Slide 6
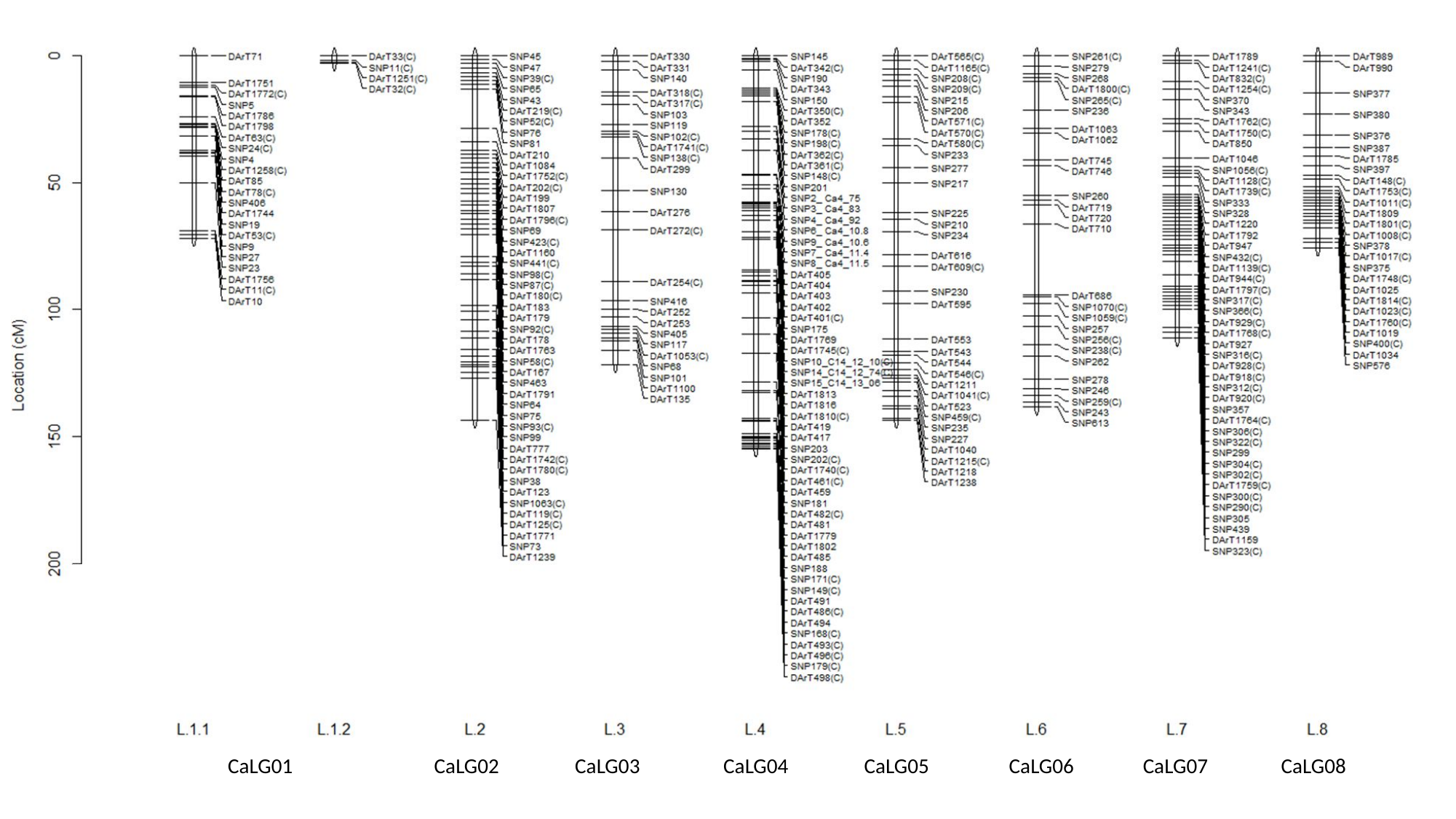

CaLG08
CaLG07
CaLG06
CaLG04
CaLG05
CaLG03
CaLG02
CaLG01

## Slide 7
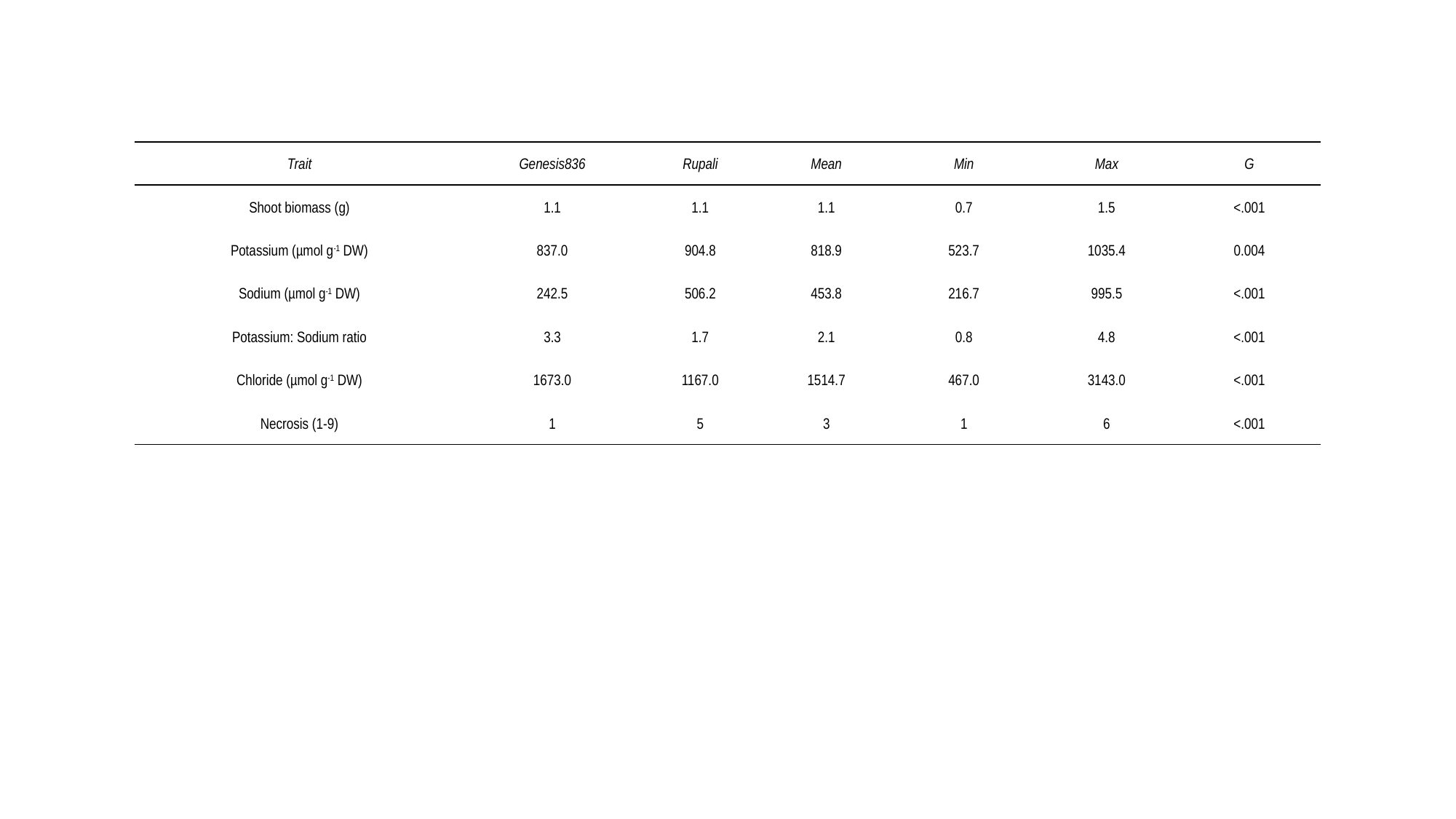

| Trait | Genesis836 | Rupali | Mean | Min | Max | G |
| --- | --- | --- | --- | --- | --- | --- |
| Shoot biomass (g) | 1.1 | 1.1 | 1.1 | 0.7 | 1.5 | <.001 |
| Potassium (µmol g-1 DW) | 837.0 | 904.8 | 818.9 | 523.7 | 1035.4 | 0.004 |
| Sodium (µmol g-1 DW) | 242.5 | 506.2 | 453.8 | 216.7 | 995.5 | <.001 |
| Potassium: Sodium ratio | 3.3 | 1.7 | 2.1 | 0.8 | 4.8 | <.001 |
| Chloride (µmol g-1 DW) | 1673.0 | 1167.0 | 1514.7 | 467.0 | 3143.0 | <.001 |
| Necrosis (1-9) | 1 | 5 | 3 | 1 | 6 | <.001 |

## Slide 8
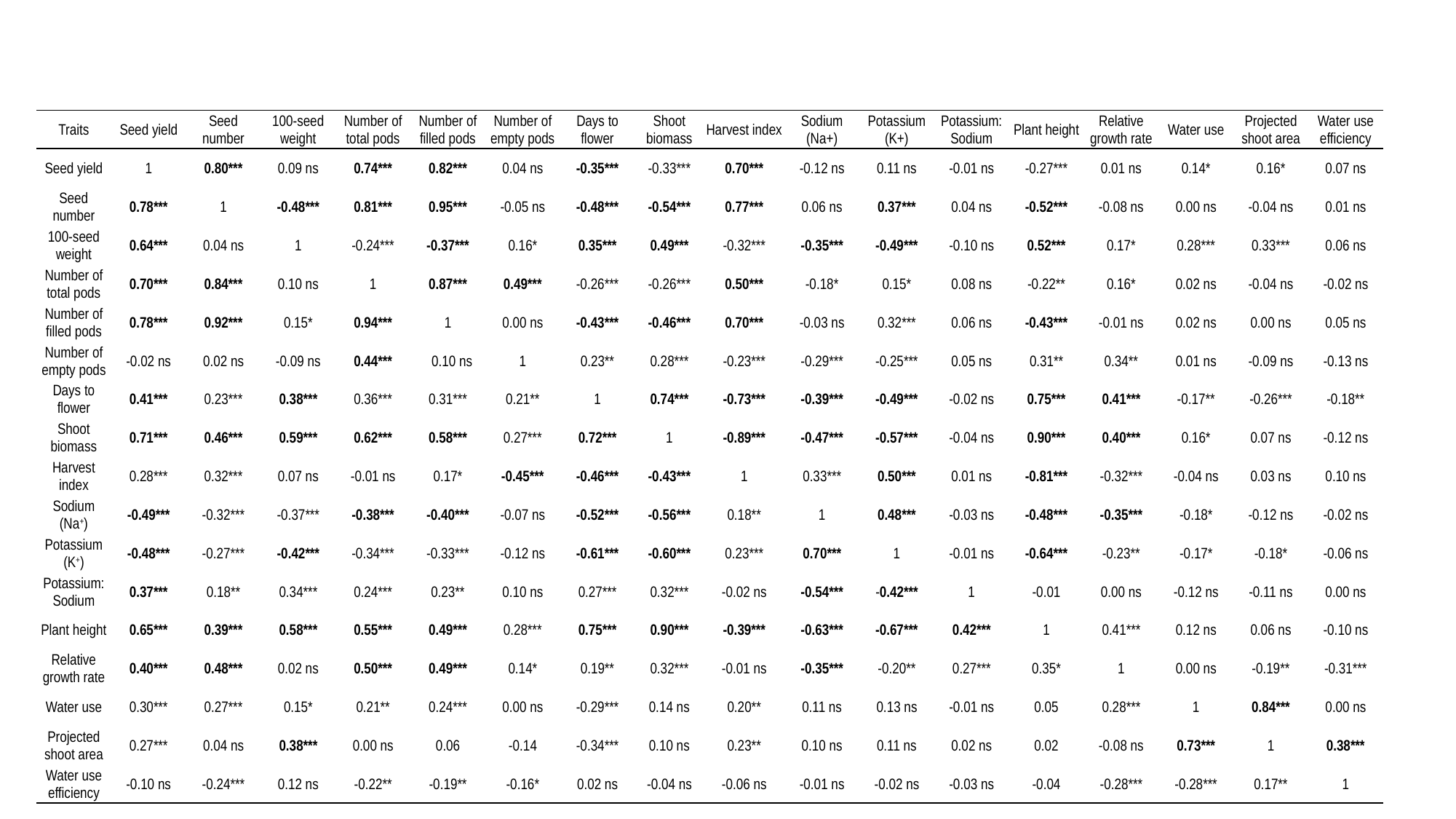

| Traits | Seed yield | Seed number | 100-seed weight | Number of total pods | Number of filled pods | Number of empty pods | Days to flower | Shoot biomass | Harvest index | Sodium (Na+) | Potassium (K+) | Potassium: Sodium | Plant height | Relative growth rate | Water use | Projected shoot area | Water use efficiency |
| --- | --- | --- | --- | --- | --- | --- | --- | --- | --- | --- | --- | --- | --- | --- | --- | --- | --- |
| Seed yield | 1 | 0.80\*\*\* | 0.09 ns | 0.74\*\*\* | 0.82\*\*\* | 0.04 ns | -0.35\*\*\* | -0.33\*\*\* | 0.70\*\*\* | -0.12 ns | 0.11 ns | -0.01 ns | -0.27\*\*\* | 0.01 ns | 0.14\* | 0.16\* | 0.07 ns |
| Seed number | 0.78\*\*\* | 1 | -0.48\*\*\* | 0.81\*\*\* | 0.95\*\*\* | -0.05 ns | -0.48\*\*\* | -0.54\*\*\* | 0.77\*\*\* | 0.06 ns | 0.37\*\*\* | 0.04 ns | -0.52\*\*\* | -0.08 ns | 0.00 ns | -0.04 ns | 0.01 ns |
| 100-seed weight | 0.64\*\*\* | 0.04 ns | 1 | -0.24\*\*\* | -0.37\*\*\* | 0.16\* | 0.35\*\*\* | 0.49\*\*\* | -0.32\*\*\* | -0.35\*\*\* | -0.49\*\*\* | -0.10 ns | 0.52\*\*\* | 0.17\* | 0.28\*\*\* | 0.33\*\*\* | 0.06 ns |
| Number of total pods | 0.70\*\*\* | 0.84\*\*\* | 0.10 ns | 1 | 0.87\*\*\* | 0.49\*\*\* | -0.26\*\*\* | -0.26\*\*\* | 0.50\*\*\* | -0.18\* | 0.15\* | 0.08 ns | -0.22\*\* | 0.16\* | 0.02 ns | -0.04 ns | -0.02 ns |
| Number of filled pods | 0.78\*\*\* | 0.92\*\*\* | 0.15\* | 0.94\*\*\* | 1 | 0.00 ns | -0.43\*\*\* | -0.46\*\*\* | 0.70\*\*\* | -0.03 ns | 0.32\*\*\* | 0.06 ns | -0.43\*\*\* | -0.01 ns | 0.02 ns | 0.00 ns | 0.05 ns |
| Number of empty pods | -0.02 ns | 0.02 ns | -0.09 ns | 0.44\*\*\* | 0.10 ns | 1 | 0.23\*\* | 0.28\*\*\* | -0.23\*\*\* | -0.29\*\*\* | -0.25\*\*\* | 0.05 ns | 0.31\*\* | 0.34\*\* | 0.01 ns | -0.09 ns | -0.13 ns |
| Days to flower | 0.41\*\*\* | 0.23\*\*\* | 0.38\*\*\* | 0.36\*\*\* | 0.31\*\*\* | 0.21\*\* | 1 | 0.74\*\*\* | -0.73\*\*\* | -0.39\*\*\* | -0.49\*\*\* | -0.02 ns | 0.75\*\*\* | 0.41\*\*\* | -0.17\*\* | -0.26\*\*\* | -0.18\*\* |
| Shoot biomass | 0.71\*\*\* | 0.46\*\*\* | 0.59\*\*\* | 0.62\*\*\* | 0.58\*\*\* | 0.27\*\*\* | 0.72\*\*\* | 1 | -0.89\*\*\* | -0.47\*\*\* | -0.57\*\*\* | -0.04 ns | 0.90\*\*\* | 0.40\*\*\* | 0.16\* | 0.07 ns | -0.12 ns |
| Harvest index | 0.28\*\*\* | 0.32\*\*\* | 0.07 ns | -0.01 ns | 0.17\* | -0.45\*\*\* | -0.46\*\*\* | -0.43\*\*\* | 1 | 0.33\*\*\* | 0.50\*\*\* | 0.01 ns | -0.81\*\*\* | -0.32\*\*\* | -0.04 ns | 0.03 ns | 0.10 ns |
| Sodium (Na+) | -0.49\*\*\* | -0.32\*\*\* | -0.37\*\*\* | -0.38\*\*\* | -0.40\*\*\* | -0.07 ns | -0.52\*\*\* | -0.56\*\*\* | 0.18\*\* | 1 | 0.48\*\*\* | -0.03 ns | -0.48\*\*\* | -0.35\*\*\* | -0.18\* | -0.12 ns | -0.02 ns |
| Potassium (K+) | -0.48\*\*\* | -0.27\*\*\* | -0.42\*\*\* | -0.34\*\*\* | -0.33\*\*\* | -0.12 ns | -0.61\*\*\* | -0.60\*\*\* | 0.23\*\*\* | 0.70\*\*\* | 1 | -0.01 ns | -0.64\*\*\* | -0.23\*\* | -0.17\* | -0.18\* | -0.06 ns |
| Potassium: Sodium | 0.37\*\*\* | 0.18\*\* | 0.34\*\*\* | 0.24\*\*\* | 0.23\*\* | 0.10 ns | 0.27\*\*\* | 0.32\*\*\* | -0.02 ns | -0.54\*\*\* | -0.42\*\*\* | 1 | -0.01 | 0.00 ns | -0.12 ns | -0.11 ns | 0.00 ns |
| Plant height | 0.65\*\*\* | 0.39\*\*\* | 0.58\*\*\* | 0.55\*\*\* | 0.49\*\*\* | 0.28\*\*\* | 0.75\*\*\* | 0.90\*\*\* | -0.39\*\*\* | -0.63\*\*\* | -0.67\*\*\* | 0.42\*\*\* | 1 | 0.41\*\*\* | 0.12 ns | 0.06 ns | -0.10 ns |
| Relative growth rate | 0.40\*\*\* | 0.48\*\*\* | 0.02 ns | 0.50\*\*\* | 0.49\*\*\* | 0.14\* | 0.19\*\* | 0.32\*\*\* | -0.01 ns | -0.35\*\*\* | -0.20\*\* | 0.27\*\*\* | 0.35\* | 1 | 0.00 ns | -0.19\*\* | -0.31\*\*\* |
| Water use | 0.30\*\*\* | 0.27\*\*\* | 0.15\* | 0.21\*\* | 0.24\*\*\* | 0.00 ns | -0.29\*\*\* | 0.14 ns | 0.20\*\* | 0.11 ns | 0.13 ns | -0.01 ns | 0.05 | 0.28\*\*\* | 1 | 0.84\*\*\* | 0.00 ns |
| Projected shoot area | 0.27\*\*\* | 0.04 ns | 0.38\*\*\* | 0.00 ns | 0.06 | -0.14 | -0.34\*\*\* | 0.10 ns | 0.23\*\* | 0.10 ns | 0.11 ns | 0.02 ns | 0.02 | -0.08 ns | 0.73\*\*\* | 1 | 0.38\*\*\* |
| Water use efficiency | -0.10 ns | -0.24\*\*\* | 0.12 ns | -0.22\*\* | -0.19\*\* | -0.16\* | 0.02 ns | -0.04 ns | -0.06 ns | -0.01 ns | -0.02 ns | -0.03 ns | -0.04 | -0.28\*\*\* | -0.28\*\*\* | 0.17\*\* | 1 |

## Slide 9
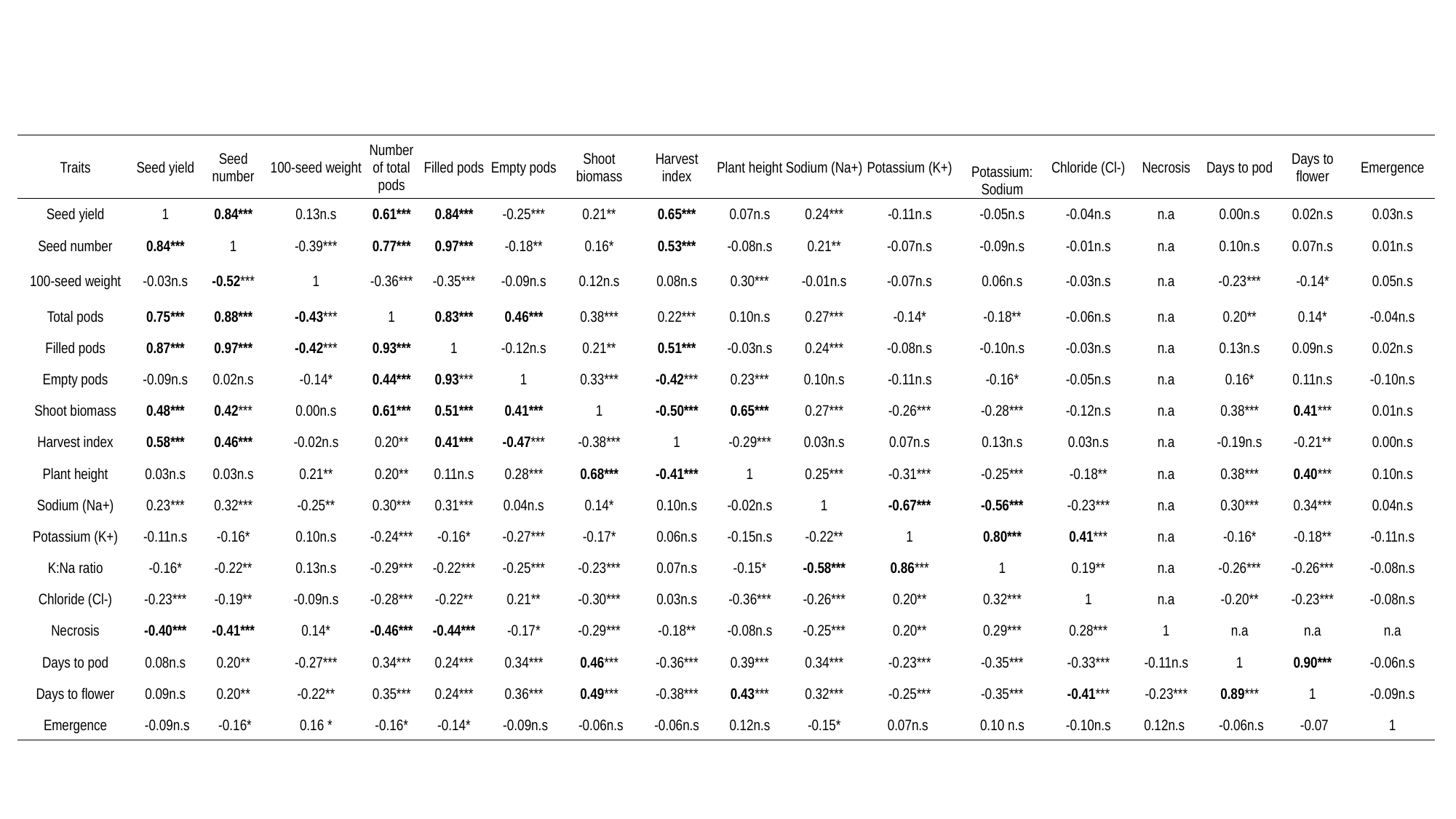

| Traits | Seed yield | Seed number | 100-seed weight | Number of total pods | Filled pods | Empty pods | Shoot biomass | Harvest index | Plant height | Sodium (Na+) | Potassium (K+) | Potassium: Sodium | Chloride (Cl-) | Necrosis | Days to pod | Days to flower | Emergence |
| --- | --- | --- | --- | --- | --- | --- | --- | --- | --- | --- | --- | --- | --- | --- | --- | --- | --- |
| Seed yield | 1 | 0.84\*\*\* | 0.13n.s | 0.61\*\*\* | 0.84\*\*\* | -0.25\*\*\* | 0.21\*\* | 0.65\*\*\* | 0.07n.s | 0.24\*\*\* | -0.11n.s | -0.05n.s | -0.04n.s | n.a | 0.00n.s | 0.02n.s | 0.03n.s |
| Seed number | 0.84\*\*\* | 1 | -0.39\*\*\* | 0.77\*\*\* | 0.97\*\*\* | -0.18\*\* | 0.16\* | 0.53\*\*\* | -0.08n.s | 0.21\*\* | -0.07n.s | -0.09n.s | -0.01n.s | n.a | 0.10n.s | 0.07n.s | 0.01n.s |
| 100-seed weight | -0.03n.s | -0.52\*\*\* | 1 | -0.36\*\*\* | -0.35\*\*\* | -0.09n.s | 0.12n.s | 0.08n.s | 0.30\*\*\* | -0.01n.s | -0.07n.s | 0.06n.s | -0.03n.s | n.a | -0.23\*\*\* | -0.14\* | 0.05n.s |
| Total pods | 0.75\*\*\* | 0.88\*\*\* | -0.43\*\*\* | 1 | 0.83\*\*\* | 0.46\*\*\* | 0.38\*\*\* | 0.22\*\*\* | 0.10n.s | 0.27\*\*\* | -0.14\* | -0.18\*\* | -0.06n.s | n.a | 0.20\*\* | 0.14\* | -0.04n.s |
| Filled pods | 0.87\*\*\* | 0.97\*\*\* | -0.42\*\*\* | 0.93\*\*\* | 1 | -0.12n.s | 0.21\*\* | 0.51\*\*\* | -0.03n.s | 0.24\*\*\* | -0.08n.s | -0.10n.s | -0.03n.s | n.a | 0.13n.s | 0.09n.s | 0.02n.s |
| Empty pods | -0.09n.s | 0.02n.s | -0.14\* | 0.44\*\*\* | 0.93\*\*\* | 1 | 0.33\*\*\* | -0.42\*\*\* | 0.23\*\*\* | 0.10n.s | -0.11n.s | -0.16\* | -0.05n.s | n.a | 0.16\* | 0.11n.s | -0.10n.s |
| Shoot biomass | 0.48\*\*\* | 0.42\*\*\* | 0.00n.s | 0.61\*\*\* | 0.51\*\*\* | 0.41\*\*\* | 1 | -0.50\*\*\* | 0.65\*\*\* | 0.27\*\*\* | -0.26\*\*\* | -0.28\*\*\* | -0.12n.s | n.a | 0.38\*\*\* | 0.41\*\*\* | 0.01n.s |
| Harvest index | 0.58\*\*\* | 0.46\*\*\* | -0.02n.s | 0.20\*\* | 0.41\*\*\* | -0.47\*\*\* | -0.38\*\*\* | 1 | -0.29\*\*\* | 0.03n.s | 0.07n.s | 0.13n.s | 0.03n.s | n.a | -0.19n.s | -0.21\*\* | 0.00n.s |
| Plant height | 0.03n.s | 0.03n.s | 0.21\*\* | 0.20\*\* | 0.11n.s | 0.28\*\*\* | 0.68\*\*\* | -0.41\*\*\* | 1 | 0.25\*\*\* | -0.31\*\*\* | -0.25\*\*\* | -0.18\*\* | n.a | 0.38\*\*\* | 0.40\*\*\* | 0.10n.s |
| Sodium (Na+) | 0.23\*\*\* | 0.32\*\*\* | -0.25\*\* | 0.30\*\*\* | 0.31\*\*\* | 0.04n.s | 0.14\* | 0.10n.s | -0.02n.s | 1 | -0.67\*\*\* | -0.56\*\*\* | -0.23\*\*\* | n.a | 0.30\*\*\* | 0.34\*\*\* | 0.04n.s |
| Potassium (K+) | -0.11n.s | -0.16\* | 0.10n.s | -0.24\*\*\* | -0.16\* | -0.27\*\*\* | -0.17\* | 0.06n.s | -0.15n.s | -0.22\*\* | 1 | 0.80\*\*\* | 0.41\*\*\* | n.a | -0.16\* | -0.18\*\* | -0.11n.s |
| K:Na ratio | -0.16\* | -0.22\*\* | 0.13n.s | -0.29\*\*\* | -0.22\*\*\* | -0.25\*\*\* | -0.23\*\*\* | 0.07n.s | -0.15\* | -0.58\*\*\* | 0.86\*\*\* | 1 | 0.19\*\* | n.a | -0.26\*\*\* | -0.26\*\*\* | -0.08n.s |
| Chloride (Cl-) | -0.23\*\*\* | -0.19\*\* | -0.09n.s | -0.28\*\*\* | -0.22\*\* | 0.21\*\* | -0.30\*\*\* | 0.03n.s | -0.36\*\*\* | -0.26\*\*\* | 0.20\*\* | 0.32\*\*\* | 1 | n.a | -0.20\*\* | -0.23\*\*\* | -0.08n.s |
| Necrosis | -0.40\*\*\* | -0.41\*\*\* | 0.14\* | -0.46\*\*\* | -0.44\*\*\* | -0.17\* | -0.29\*\*\* | -0.18\*\* | -0.08n.s | -0.25\*\*\* | 0.20\*\* | 0.29\*\*\* | 0.28\*\*\* | 1 | n.a | n.a | n.a |
| Days to pod | 0.08n.s | 0.20\*\* | -0.27\*\*\* | 0.34\*\*\* | 0.24\*\*\* | 0.34\*\*\* | 0.46\*\*\* | -0.36\*\*\* | 0.39\*\*\* | 0.34\*\*\* | -0.23\*\*\* | -0.35\*\*\* | -0.33\*\*\* | -0.11n.s | 1 | 0.90\*\*\* | -0.06n.s |
| Days to flower | 0.09n.s | 0.20\*\* | -0.22\*\* | 0.35\*\*\* | 0.24\*\*\* | 0.36\*\*\* | 0.49\*\*\* | -0.38\*\*\* | 0.43\*\*\* | 0.32\*\*\* | -0.25\*\*\* | -0.35\*\*\* | -0.41\*\*\* | -0.23\*\*\* | 0.89\*\*\* | 1 | -0.09n.s |
| Emergence | -0.09n.s | -0.16\* | 0.16 \* | -0.16\* | -0.14\* | -0.09n.s | -0.06n.s | -0.06n.s | 0.12n.s | -0.15\* | 0.07n.s | 0.10 n.s | -0.10n.s | 0.12n.s | -0.06n.s | -0.07 | 1 |

## Slide 10
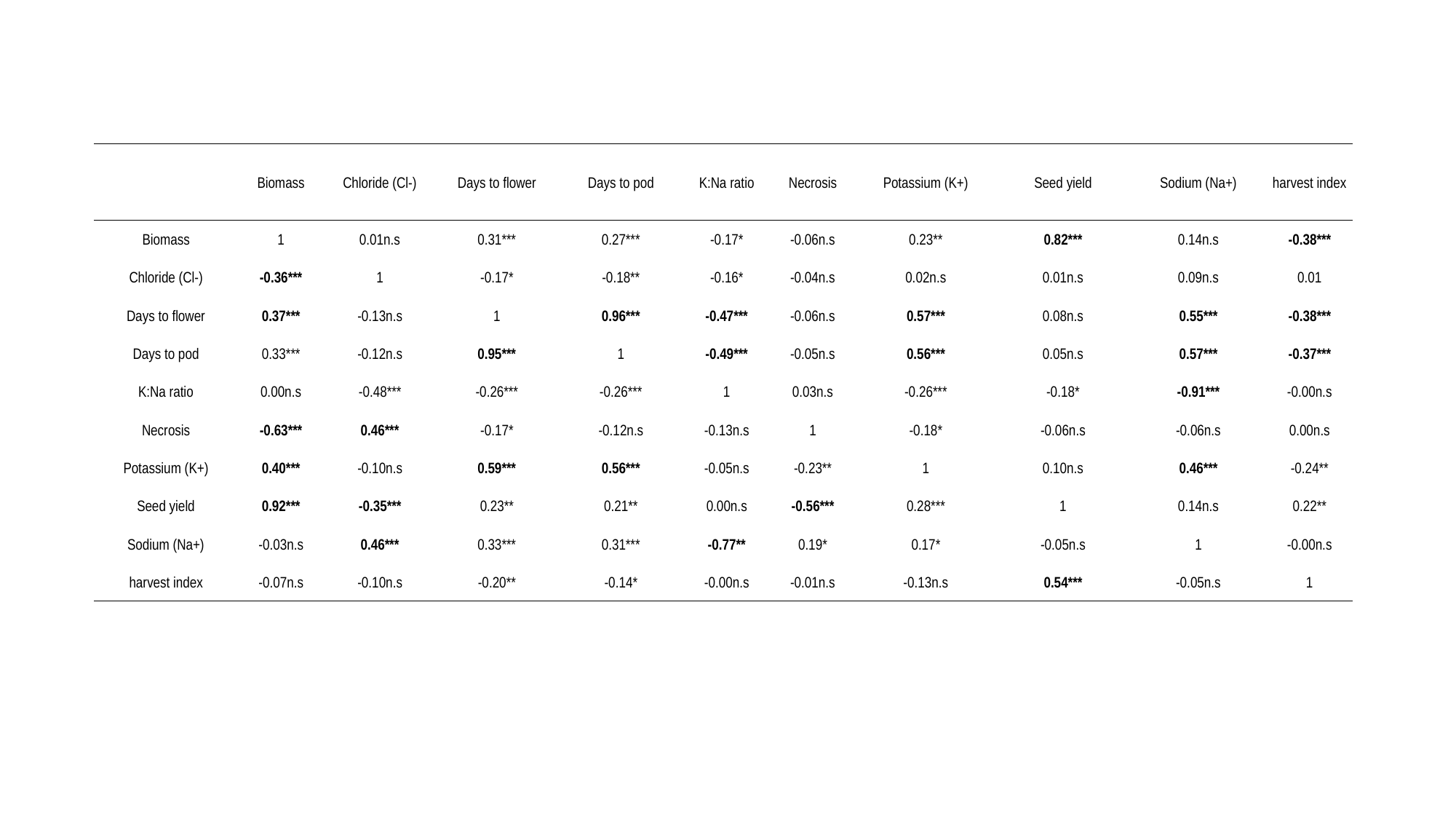

| | Biomass | Chloride (Cl-) | Days to flower | Days to pod | K:Na ratio | Necrosis | Potassium (K+) | Seed yield | Sodium (Na+) | harvest index |
| --- | --- | --- | --- | --- | --- | --- | --- | --- | --- | --- |
| Biomass | 1 | 0.01n.s | 0.31\*\*\* | 0.27\*\*\* | -0.17\* | -0.06n.s | 0.23\*\* | 0.82\*\*\* | 0.14n.s | -0.38\*\*\* |
| Chloride (Cl-) | -0.36\*\*\* | 1 | -0.17\* | -0.18\*\* | -0.16\* | -0.04n.s | 0.02n.s | 0.01n.s | 0.09n.s | 0.01 |
| Days to flower | 0.37\*\*\* | -0.13n.s | 1 | 0.96\*\*\* | -0.47\*\*\* | -0.06n.s | 0.57\*\*\* | 0.08n.s | 0.55\*\*\* | -0.38\*\*\* |
| Days to pod | 0.33\*\*\* | -0.12n.s | 0.95\*\*\* | 1 | -0.49\*\*\* | -0.05n.s | 0.56\*\*\* | 0.05n.s | 0.57\*\*\* | -0.37\*\*\* |
| K:Na ratio | 0.00n.s | -0.48\*\*\* | -0.26\*\*\* | -0.26\*\*\* | 1 | 0.03n.s | -0.26\*\*\* | -0.18\* | -0.91\*\*\* | -0.00n.s |
| Necrosis | -0.63\*\*\* | 0.46\*\*\* | -0.17\* | -0.12n.s | -0.13n.s | 1 | -0.18\* | -0.06n.s | -0.06n.s | 0.00n.s |
| Potassium (K+) | 0.40\*\*\* | -0.10n.s | 0.59\*\*\* | 0.56\*\*\* | -0.05n.s | -0.23\*\* | 1 | 0.10n.s | 0.46\*\*\* | -0.24\*\* |
| Seed yield | 0.92\*\*\* | -0.35\*\*\* | 0.23\*\* | 0.21\*\* | 0.00n.s | -0.56\*\*\* | 0.28\*\*\* | 1 | 0.14n.s | 0.22\*\* |
| Sodium (Na+) | -0.03n.s | 0.46\*\*\* | 0.33\*\*\* | 0.31\*\*\* | -0.77\*\* | 0.19\* | 0.17\* | -0.05n.s | 1 | -0.00n.s |
| harvest index | -0.07n.s | -0.10n.s | -0.20\*\* | -0.14\* | -0.00n.s | -0.01n.s | -0.13n.s | 0.54\*\*\* | -0.05n.s | 1 |
